# An autophagy assay reveals the ESCRT-III component CHMP2A as a regulator of phagophore closure

**DOI:** 10.1038/s41467-018-05254-w

**Published:** 2018-07-20

**Authors:** Yoshinori Takahashi, Haiyan He, Zhenyuan Tang, Tatsuya Hattori, Ying Liu, Megan M. Young, Jacob M. Serfass, Longgui Chen, Melat Gebru, Chong Chen, Carson A. Wills, Jennifer M. Atkinson, Han Chen, Thomas Abraham, Hong-Gang Wang

**Affiliations:** 1 Department of Pediatrics, Penn State College of Medicine, Hershey, PA 17033 USA; 2 Department of Pharmacology, Penn State College of Medicine, Hershey, PA 17033 USA; 3 Microscopy Imaging Facility, Penn State College of Medicine, Hershey, PA 17033 USA; 4 Department of Neural and Behavioral Science, Penn State College of Medicine, Hershey, PA 17033 USA

## Abstract

The mechanism of phagophore closure remains unclear due to technical limitations in distinguishing unclosed and closed autophagosomal membranes. Here, we report the HaloTag-LC3 autophagosome completion assay that specifically detects phagophores, nascent autophagosomes, and mature autophagic structures. Using this assay, we identify the endosomal sorting complexes required for transport (ESCRT)-III component CHMP2A as a critical regulator of phagophore closure. During autophagy, CHMP2A translocates to the phagophore and regulates the separation of the inner and outer autophagosomal membranes to form double-membrane autophagosomes. Consistently, inhibition of the AAA-ATPase VPS4 activity impairs autophagosome completion. The ESCRT-mediated membrane abscission appears to be a critical step in forming functional autolysosomes by preventing mislocalization of lysosome-associated membrane glycoprotein 1 to the inner autophagosomal membrane. Collectively, our work reveals a function for the ESCRT machinery in the final step of autophagosome formation and provides a useful tool for quantitative analysis of autophagosome biogenesis and maturation.

## Abstract (web summary)

During autophagy, phagophores elongate to form double-membrane vesicles but the mechanism behind their closure is unknown. Here, the authors develop an autophagy assay and find a role for the endosomal sorting complexes required for transport component CHMP2A as a phagophore closure regulator.

## Introduction

Macroautophagy (hereafter referred to as autophagy) is an intracellular catabolic process where cytoplasmic material is sequestered into double-membrane autophagosomes for lysosomal degradation^1^. A series of AuTophaGy-related (ATG) proteins coordinate the initiation, nucleation, and elongation of crescent-shaped isolation membranes or phagophores during autophagosome biogenesis^2,3^. However, how the phagophore undergoes membrane remodeling to generate the inner and outer membranes of the completed autophagosome remains far from clear^4^ and has been hindered by technical challenges associated with distinguishing unclosed and closed autophagosomal membranes^5^.

The endosomal sorting complex required for transport (ESCRT) proteins were originally identified as regulators of ubiquitinated cargo sorting into multivesicular bodies (MVBs) but have since extended to mediate reverse-topology membrane scission in a variety of cellular processes^6,7^. While ESCRTs failed to be identified in yeast screens for essential ATG genes, ESCRT defects in *C. elegans*, *Drosophila* and mammals accumulate autophagosome-like structures^8–11^. Interestingly, the topological membrane transformation that occurs during phagophore closure resembles that of ESCRT-mediated intraluminal vesicle formation^7,12,13^.

Microtubule-associated protein 1 light chain 3 (LC3) is a mammalian ortholog of yeast Atg8 that is conjugated with phosphatidylethanolamine to form LC3-II at the phagophore during autophagosome biogenesis^14,15^. Upon closure, LC3-II on the outer autophagosomal membrane (OAM) is delipidated and released to the cytosol, while LC3-II associated with the inner autophagosomal membrane (IAM) is degraded upon autophagosome-lysosome fusion^16^. Exploiting the unique topology of LC3 during autophagosome biogenesis, we here develop an autophagosome completion assay to differentiate phagophores, nascent and mature autophagosomes and explore the role of the ESCRT-mediated membrane fission machinery in phagophore closure. We demonstrate that proper autophagosomal membrane closure requires the ESCRT-III component CHMP2A and the AAA-ATPase Vacuolar Protein Sorting-associated 4 (VPS4) activity and that the generation of the OAM and IAM by ESCRT-mediated membrane abscission prior to lysosomal recruitment is a critical step in the formation of functional autolysosomes.

## Results

### Development of the HT-LC3 autophagosome completion assay

**Fig. 1:**
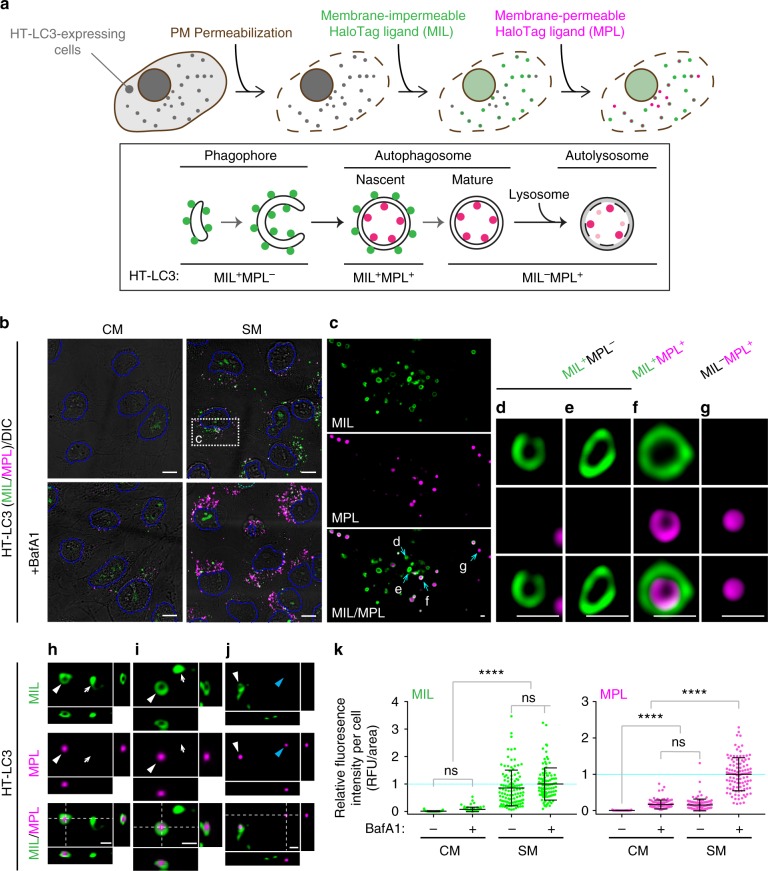
The HaloTag-LC3 autophagosome completion assay distinguishes unclosed and closed autophagosomal membranes. **a** Schematic strategy of the HT-LC3 autophagosome completion assay. The assay is performed by the following procedures: step 1, after the induction of autophagy, HT-LC3-expressing cells are treated with cholesterol-complexing agents including recombinant perfringolysin (rPFO/XF-MPM) or digitonin to permeabilize the plasma membrane (PM) and release HT-LC3-I from the cytosol; step 2, cells are incubated with a saturating dose of membrane-impermeable HT ligand (MIL) to stain membrane-bound HT-LC3-II that is accessible to the cytosol (MIL also diffuses into nucleus and stains nuclear LC3); step 3, cells are incubated with membrane-permeable HT ligand (MPL) to stain LC3-II that is sequestered within membranes. **b**–**j** HT-LC3 U-2 OS cells were incubated in starvation medium (SM) or control complete medium (CM) in the presence or absence of 100 nM BafA1 for 4 h (**b**–**g**) or starved for 3 h (**h**–**j**) and subjected to the HT-LC3 autophagosome completion assay followed by confocal microscopy (**b**–**g**) and 3D-deconvolution fluorescence microscopy (**h**–**j**). LC3 signals on the phagophore or the outer autophagosomal membrane, and in the autophagosome lumen were stained using Alexa Fluor 488 (AF488)-conjugated MIL and tetramethylrhodamine (TMR)-conjugated MPL, respectively. Magnified images of the boxed area in (**b**) and arrow-indicated areas in (**c**) are shown in (**c**) and (**d**–**g**), respectively. In (**h**–**j**), xz and yz images at the dash-lined area in Supplementary Fig. 1c are shown to the right and bottom, respectively; arrows, white arrowheads and blue arrowheads indicate MIL^+^MPL^−^, MIL^+^MPL^+^, and MIL^−^MPL^+^ structures, respectively. The scale bars represent 10 μm and 1 μm in (**b**) and (**c**–**j**), respectively. (**k**) The cytoplasmic fluorescence intensities of MIL and MPL in each cell in (**b**) were quantified and normalized to the respective mean fluorescence intensities of the cells starved in the presence of BafA1 (*n* > 100). Data shown are representative of three independent experiments. Statistical significance was determined by one-way ANOVA followed by Tukey’s multiple comparison test. All values are mean ± SD. ns not significant; ****p ≤ 0.0001

To distinguish unclosed and closed autophagosomal membranes, we utilized the reporter HaloTag-LC3 (HT-LC3) in combination with membrane-impermeable Alexa Fluor (AF) 488 or AF660 HaloTag ligand (MIL) and membrane-permeable tetramethylrhodamine HaloTag ligand (MPL) (Fig. 1a). We first generated U-2 OS cells stably expressing HT-LC3 and confirmed that starvation-induced HT-LC3-I lipidation and HT-LC3-II turnover were comparable to endogenous LC3 (Supplementary Fig. 1a). To perform the autophagosome completion assay, cells were starved to induce autophagy followed by permeabilization of the plasma membrane to release cytosolic HT-LC3-I and sequential labeling of HT-LC3-II with MIL and MPL (Fig. 1a). We verified that, while cytosolic HT signals were lost during permeabilization, HT-labeled autophagic structures were retained in the cytoplasmic region throughout the assay process (Supplementary Fig. 1b). The specificity of each HaloTag ligand and the saturation of phagophore- and OAM-associated HT-LC3-II by the initial MIL labeling were verified during assay optimization (Supplementary Fig. 1c, d). As expected, three populations of HT-LC3-II structures were detected in the cytoplasmic region of starved cells: (1) MIL^+^MPL^−^, representing phagophores, (2) MIL^+^MPL^+^, representing nascent autophagosomes, and (3) MIL^−^MPL^+^, representing mature autophagosomes, amphisomes, and autolysosomes (Fig. 1b, c). Moreover, the MIL^+^MPL^−^ signals displayed cup- or oval-shaped structures (Fig.1d, e; arrows in h, i) in agreement with phagophore morphology^17^, while MIL^+^MPL^+^ signals formed nascent autophagosome-like structures in which MIL signals (OAM-associated HT-LC3-II) surrounded MPL signals (IAM-associated HT-LC3-II) (Fig. 1f; white arrowheads in h–j) and MIL^−^MPL^+^ puncta were consistent with mature autophagosomal structures in which OAM-associated LC3-II has been delipidated (Fig. 1g; blue arrowheads in j). While nutrient starvation significantly increased cytoplasmic MIL and MPL HT-LC3 signals, only MPL signals were strongly accumulated upon lysosomal inhibition (Fig. 1k) to indicate lysosomal degradation of IAM- but not OAM-associated HT-LC3-II. Similar results were obtained in HeLa cells stably expressing HT-LC3 in the presence or absence of BafA1 or lysosomal protease inhibitors (PIs) (Supplementary Fig. 1e).

**Fig. 2:**
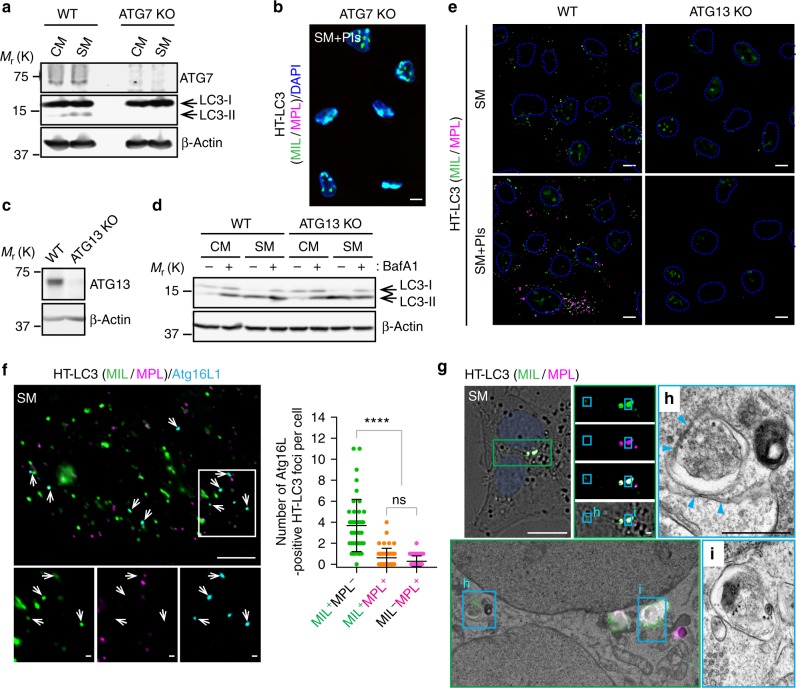
The cytoplasmic HT-LC3 positive foci specifically represent autophagic structures. **a** ATG7-deficient U-2 OS cells were generated using the Crispr-Cas9 gene editing system and subjected to starvation for 3 h followed by immunoblotting using the indicated antibodies. **b** ATG7-deficient HT-LC3 U-2 OS cells were starved in the presence of lysosomal protease inhibitors (PIs) and subjected to the HT-LC3 autophagosome completion assay followed by deconvolution fluorescence microscopy. **c**, **d** ATG13-deficient U-2 OS cells were generated using the Crispr-Cas9 gene editing system. The resultant cells were subjected to immunoblotting using the indicated antibodies (**c**) or starved in the presence or absence of 100 nM BafA1 followed by immunoblot analysis (**d**). **e** ATG13-deficient HT-LC3 U-2 OS cells were starved in the presence or absence of PIs and subjected to the HT-LC3 autophagosome completion assay followed by confocal microscopy. **f**, **g** HT-LC3 U-2 OS cells were starved for 2 h, stained with MIL and MPL, and subjected to immunofluorescence microscopy using anti-Atg16L antibody (**f**) or correlative electron microscopy (**g**). **h**, **i** The electron microscopy images of the boxed areas in (**g**). Arrows in (**f**) and arrowheads in (**h**) indicate ATG16L-positive MIL^+^MPL^−^ foci and phagophore-associated endoplasmic reticulum (ER), respectively. In (**f**), the number of ATG16L-positive MIL^+^MPL^−^, MIL^+^MPL^+^ and MIL^−^MPL^+^ foci per cell was quantified and shown (*n* = 45 from two independent experiments). Statistical significance was determined by one-way ANOVA followed by Tukey’s multiple comparison test. All values are mean ± SD. ns not significant; ****p ≤ 0.0001. The scale bars represent 10 μm and 1 μm in (**h**) and the magnified images in (**f, g**)

LC3 is an aggregation-prone protein that can be incorporated into protein aggregates independent of autophagy^18^. To confirm that the HT-LC3-positive foci represent autophagic structures, we generated LC3-I lipidation-defective U-2 OS cells by disrupting the *ATG7* gene (Fig. 2a) and performed the HT-LC3 autophagosome completion assay. We found that the cytoplasmic region of Atg7-depleted cells was not labeled by MIL or MPL, thus demonstrating the successful removal of soluble HT-LC3-I and the specificity of HaloTag ligands for membrane-bound LC3-II rather than LC3 aggregates (Fig. 2b). To exclude the possibility that HL-LC3-II localizes on non-autophagic membranes, similar experiments were performed in the absence of *ATG13*, which is dispensable for LC3-II conversion but required for autophagosome formation^19^. Indeed, knockout of *ATG13* did not affect LC3 lipidation but suppressed starvation-induced LC3-II turnover (Fig. 2c, d). Importantly, the cytoplasmic HT-LC3 foci formation was severely impaired in ATG13-deficient cells (Fig. 2e), indicating that the cytoplasmic HL-LC3 puncta are autophagosomal structures. IF staining for the phagophore marker ATG16L further validated the assay specificity, as the majority of ATG16L-positive HT-LC3 puncta were MIL^+^MPL^−^ rather than MIL^+^MPL^+^ or MIL^-^MPL^+^ (Fig. 2f). Moreover, correlative electron microscopy confirmed that MIL^+^MPL^−^ and MIL^+^MPL^+^ signals represent ER-associated phagophores (Fig. 2g, h) and autophagosome-like structures (Fig. 2g, i), respectively. Collectively, we succeeded in developing an autophagosome completion assay that distinguishes phagophores, nascent autophagosomes, and mature autophagic vacuoles.

### Screening of ESCRTs involved in phagophore closure

The topological membrane transformation that occurs during phagophore closure resembles that of ESCRT-mediated intraluminal vesicle formation^7,12,13^. To determine if the ESCRT machinery regulates phagophore closure, we screened a siRNA library targeting 40 different ESCRT-related genes (Supplementary Table 1) using the HT-LC3 autophagosome completion assay. The screening was performed using serum-free Accell siRNA delivery medium in which autophagy was induced comparable to SM (Supplementary Fig. 2a). Among the strongest hits for the accumulation of MIL^+^MPL^−^ structures compared to siNT control were several ESCRT-III siRNAs including siCHMP2A, siCHMP3 and siCHMP7 (Supplementary Fig. 2b). Increased MIL^+^MPL^−^ signals were also detected in cells treated with targeting (CEP55, PDCD6), bridging (VPS37A), and remodeling (AURKB) ESCRT siRNAs. We next validated the screening results using independent siRNA pools targeting CHMP2A, CHMP3, CHMP7, and CEP55. Knockdown of the ESCRT components was confirmed by immunoblotting (IB) (Supplementary Fig. 3a). Consistent with the screening results, depletion of each ESCRT, but not lysosomal inhibition, resulted in a significant increase in MIL^+^ immature autophagosomal membranes under starved conditions (Supplementary Fig. 3b) to suggest a role of the ESCRT machinery in phagophore closure. Among the hits, siCHMP2A displayed the strongest accumulation of MIL signals.

### CHMP2A deficiency results in phagophore accumulation

**Fig. 3:**
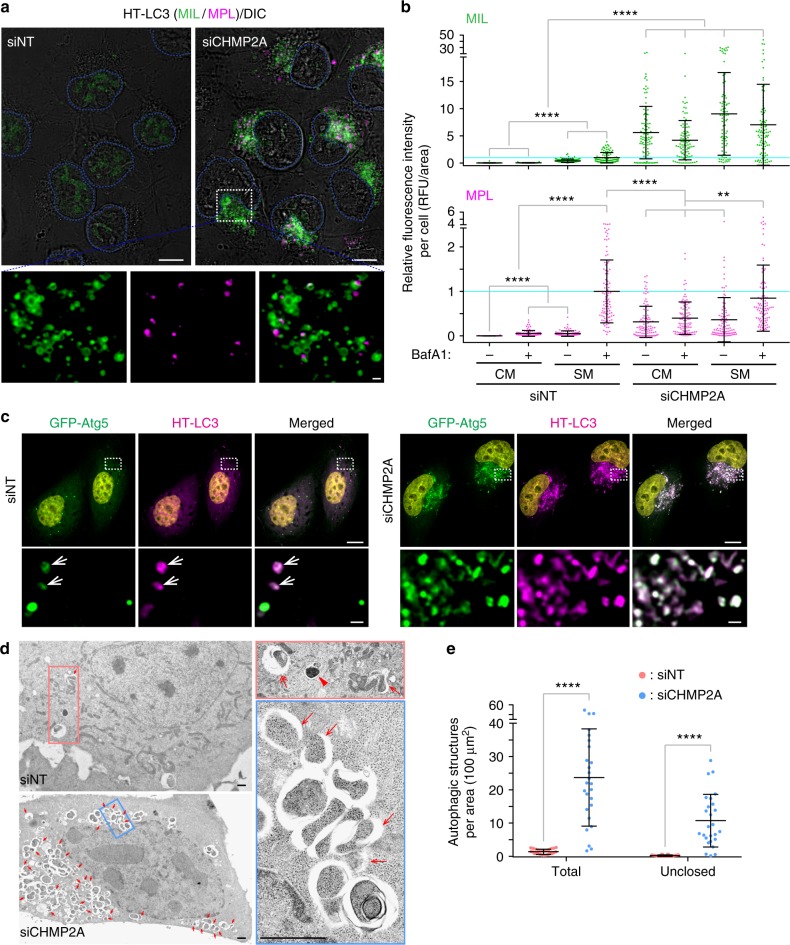
CHMP2A depletion results in the accumulation of unclosed autophagosomal membranes. HT-LC3-expressing (**a**), HT-LC3- and GFP-Atg5-expressing (**c**), or parental wild-type (**d**) U-2 OS cells were transfected with the indicated ON-TARGETplus SMART Pool siRNAs for 48 h. **a** Cells were subjected to the HT-LC3 autophagosome completion assay followed by confocal microscopy. **b** Cells were incubated in CM or SM in the presence or absence of 100 nM BafA1 for 2 h and subjected to the HT-LC3 autophagosome completion assay. Representative images are shown in Supplementary Fig. 4b. The cytoplasmic fluorescence intensities of MIL and MPL in each cell were quantified and normalized to the respective mean fluorescence intensities of control siNT-transfected cells starved in the presence of BafA1 (*n* > 100). Data shown are representative of three independent experiments. Statistical significance was determined by Kruskal–Wallis one-way ANOVA on ranks followed by Dunn’s multiple comparison test. All values are mean ± SD. **p ≤ 0.01; ****p ≤ 0.0001. **c** Cells were stained with TMR-conjugated MPL, starved for 2 h and subjected to confocal microscopy. **d** Cells were starved for 3 h and subjected to electron microscopy. The samples were processed in the absence of potassium ferrocyanide. Arrows, two-headed arrow, and arrowhead indicate phagophore-like (clearly opened in 2D micrographs), autophagosome-like, and autolysosome-like structures, respectively. **e** The number of total and unclosed autophagic structures per cytoplasmic area in (**d**) was quantified and shown (*n* = 26). Statistical significance was determined by two-way ANOVA followed by Sidak’s multiple comparison test. All values are mean ± SD. ****p ≤ 0.0001. The scale bars represent 10 μm and 1 μm in the magnified images

CHMP2A and 2B are the components of ESCRT-III that form capping assemblies with CHMP3 to drive membrane scission^6,20^. Our data showed that depletion of CHMP2A (Fig. 3a, b; Supplementary Fig. 4a–c) but not CHMP2B (Supplementary Fig. 5a, b) resulted in a dramatic increase in MIL^+^ immature autophagosomal membranes in both U-2 OS and HeLa cells under basal and starved conditions. Many of the MIL^+^ structures in CHMP2A-depleted cells were negative for MPL (magnified images in Fig. 3a; Supplementary Fig. 4c), indicating the accumulation of phagophores. Accumulation of LC3-positive structures by CHMP2A depletion was also observed using GFP-LC3 (Supplementary Fig. 4d) or by staining for endogenous LC3 (Supplementary Fig. 4e) to demonstrate that the phenotype is not due to ectopic expression of HT-LC3. Moreover, the accumulated LC3 structures in siCHMP2A cells contained not only an autophagic substrate p62 but also the phagophore markers ATG5, ATG2A, and ATG9A (Fig. 3c; Supplementary Fig. 6a, b); a result consistent with the failed dissociation of ATG machinery in the absence of closure^2^. We next performed electron microscopy to determine the ultrastructure of LC3-positive foci accumulated in CHMP2A-depleted cells. Consistently, depletion of CHMP2A accumulated immature autophagosomal structures, some of which appeared to be unclosed (asterisks in Supplementary Fig. 6c). To quantify this observation, a similar experiment was performed in the absence of potassium ferrocyanide, which artificially enlarges the intermembrane space of phagophores and autophagosomes^21,22^ to allow us to easily detect immature autophagic structures. As expected, a significantly increased number of unclosed autophagosomal membranes was observed by CHMP2A depletion in 2D electron micrographs (Fig. 3d, e). However, we note that due to the limitation of 2D electron microscopy to distinguish closed and unclosed autophagosomal membranes, the actual number of ‘unclosed’ membranes is likely more than that of shown. Collectively, these results demonstrate that CHMP2A depletion results in the accumulation of phagophores.

### CHMP2A is required for autophagy

**Fig. 4:**
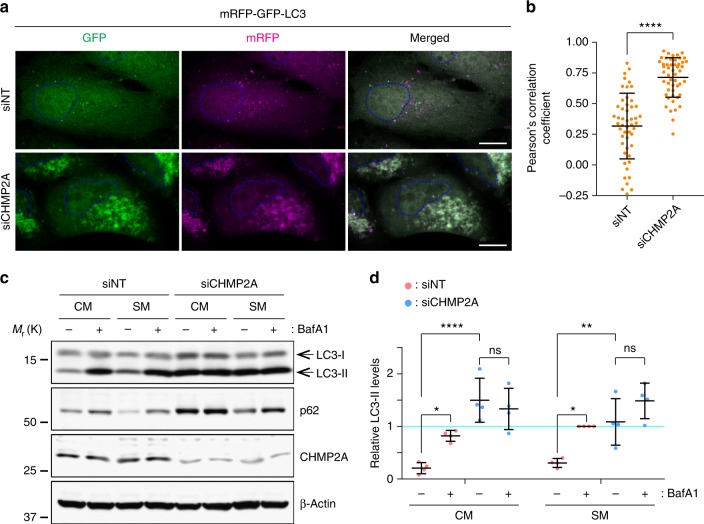
CHMP2A depletion impairs autophagic flux. **a**, **c** U-2 OS cells were transfected with the indicated ON-TARGETplus SMART Pool siRNAs. **a** Eight hours after transfection, cells were transduced with lentiviruses encoding mRFP-GFP-LC3, cultured for 40 h, incubated in CM or SM for 3 h, and subjected to confocal microscopy. The images shown are representative from the starved groups shown in Supplementary Fig. 6d. The scale bars represent 10 μm. **b** The acidification of autophagic structures in (**a**) was assessed by Pearson’s correlation coefficient. Fifty cells from each group were analyzed. Statistical significance was determined by Mann–Whitney nonparametric *t*-test. All values are mean ± SD. ****p ≤ 0.0001. **c** Forty-eight hours after transfection, cells were incubated in CM or SM in the presence or absence of 100 nM BafA1 for 3 h and subjected to immunoblotting using the indicated antibodies. Representative blots from four independent experiments are shown. **d** The LC3-II levels relative to respective β-actin in (**c**) were quantified and normalized to the value of starved, siNT-transfected cells. Statistical significance was determined by two-way ANOVA followed by Tukey’s multiple comparison test. All values are mean ± SD. ns not significant; *p ≤ 0.05; **p ≤ 0.01; ****p ≤ 0.0001

The increase in LC3-II-positive foci is attributed either to the inhibition of autophagic flux or to the promotion of autophagosome biogenesis^5^. To determine the effect of CHMP2A depletion on autophagic flux, we first performed the tandem fluorescent-tagged LC3 assay^23^. This assay is based on the difference in p*Ka* values between RFP and GFP to distinguish non-degradative (GFP^+^RFP^+^) and degradative (GFP^−^RFP^+^) autophagic structures. While starvation induced both GFP^+^RFP^+^ and GFP^−^RFP^+^ structures in control cells, CHMP2A depletion resulted in the accumulation of GFP^+^RFP^+^, but not GFP^−^RFP^+^, structures under both starved and non-starved conditions, indicating the impairment of autophagic flux (Fig. 4a, b; Supplementary Fig. 6d). Consistently, both basal and starvation-induced lysosomal turnover of LC3-II was found to be impaired by the depletion of CHMP2A (Fig. 4c, d), but not CHMP2B (Supplementary Fig. 5c). These results indicate an indispensable role of CHMP2A in basal and induced autophagy.

### CHMP2A translocates to the phagophore during autophagy

**Fig. 5:**
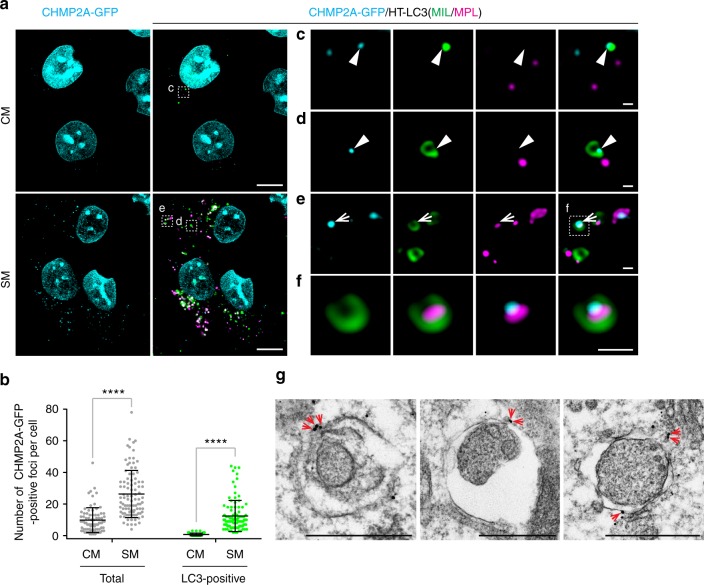
CHMP2A translocates to the phagophore in response to nutrient starvation. **a** HT-LC3 U-2 OS cells were transduced with lentiviruses encoding CHMP2A-GFP and cultured for 6 days. The resultant cells stably expressing CHMP2A-GFP were incubated in CM or SM for 2 h and subjected to the HT-LC3 autophagosome completion assay using AF660-conjugated MIL and TMR-conjugated MPL followed by confocal microscopy. **b** The number of total and LC3-associated CHMP2A-GFP-positive foci per cell in (**a**) was quantified and shown as dot plots (*n* > 80). Statistical significance was determined using two-way ANOVA with Sidak’s multiple comparison test. All values are mean ± SD. ****p ≤ 0.0001. Data shown are representative of two independent experiments. **c**–**f** Magnified images in the boxed areas in (**a**) are shown. Arrowheads and arrows indicate colocalization of CHMP2A with a MIL^+^MPL^−^ phagophore-like and a MIL^+^MPL^+^ immature autophagosome-like structures. **g** CHMP2A-GFP-expressing U-2 OS cells were starved for 2 h and subjected to immunoelectron microscopy using anti-GFP antibody. The scale bars represent 10 μm, 1 μm, and 500 nm in (**a**), (**c**–**f**), and (**g**)

We next determined if CHMP2A localizes on the phagophore during autophagy. As the commercially available CHMP2A antibodies failed to show the specificity in our hands (Supplementary Fig. 7), we generated a GFP-tagged CHMP2A. We found that overexpression of CHMP2A-GFP accumulated MIL^+^MPL^−^ structures in a similar manner to CHMP2A depletion (Supplementary Fig. 8), which is in agreement with the previous observation that overexpression of several ESCRT-III components acts as a dominant-negative to inhibit MVB sorting^24^. We thus generated HT-LC3 U-2 OS cells that stably express CHMP2A-GFP but do not accumulate autophagosomal membranes under basal conditions (Fig. 5a). We found that nutrient starvation induced CHMP2A-GFP foci formation in the cytoplasm with about half of the signals positive for LC3 (Fig. 5b). Importantly, CHMP2A signals were detected adjacent to LC3-positive puncta and not in the MPL^+^ autophagosomal lumen (Fig. 5c–f) with a subset of CHMP2A signals located at the edge of phagophore-like structures (arrowheads in Fig. 5c, d) as well as nascent autophagosome-like structures (arrows in Fig. 5e, f). Consistently, CHMP2A signals were detected on phagophore/immature autophagosome-like structures by immunoelectron microscopy (IEM) (arrows in Fig. 5g). Collectively, our data demonstrate that CHMP2A translocates to the phagophore during autophagy.

### Inhibition of VPS4 impairs autophagosome completion

**Fig. 6:**
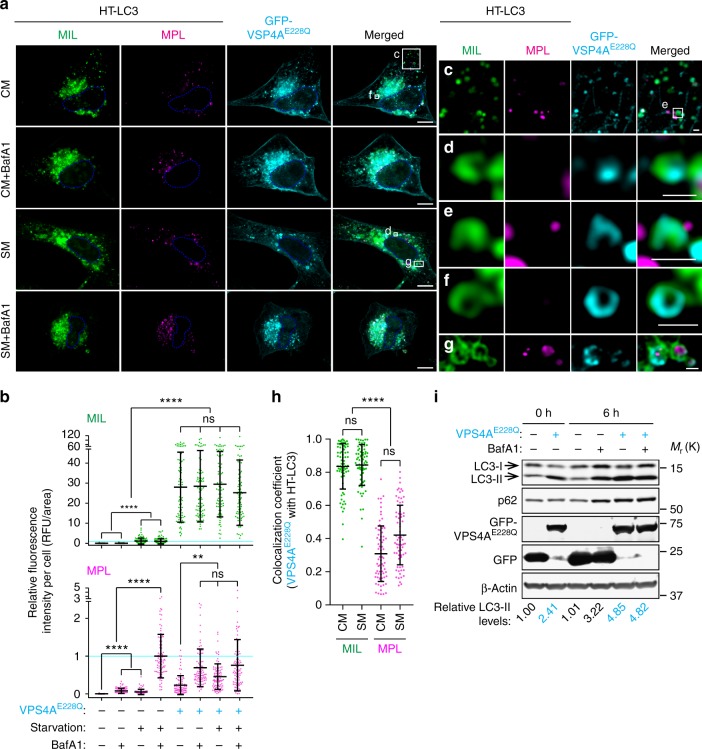
Inhibition of VPS4 accumulates MIL^+^MPL^−^ phagophores and impairs autophagic flux. HT-LC3 U-2 OS (**a**–**h**) and wild-type U2-OS cells (**i**) were transfected with GFP-VPS4A^E228Q^ or control GFP. **a** Twelve hours after the transfection, cells were incubated in CM or SM for 3 h and subjected to the HT-LC3 autophagosome completion assay followed by confocal microscopy. The scale bars represent 10 μm and 1 μm in the magnified images. **b** The cytoplasmic fluorescence intensities of MIL and MPL per cell in (**a**) were quantified and normalized to the respective mean fluorescence intensities of control GFP transfected cells starved in the presence of BafA1 (*n* > 81). Data shown are representative of two independent experiments. (**c**–**g**) Magnified images in the boxed areas in (**a**) are shown. **h** Colocalization coefficient of GFP-VPS4A^E228Q^ with MIL or MPL-labeled HT-LC3 per cell in (**a**) were quantified and shown (*n* > 72). In (**b** and **h**), statistical significance was determined by Kruskal–Wallis one-way ANOVA on ranks followed by Dunn’s multiple comparison test. All values are mean ± SD. ns not significant; **p ≤ 0.01; ****p ≤ 0.0001. **i** Six hours after transfection (0 h time point), cells were incubated in the presence or absence of 100 nM BafA for 6 h and subjected to immunoblotting using the indicated antibodies. The LC3-II levels relative to respective β-actin were quantified and normalized to the value of control GFP cells at time 0

The AAA-ATPase VPS4 hydrolyzes ATP to depolymerize ESCRT-III assemblies from membranes^6,25^. As the function of VPS4 is indispensable for ESCRT-mediated membrane fission^26^, we examined if inhibition of the VPS4 activity impairs phagophore closure using the HT-LC3 autophagosome completion assay. Similar to CHMP2A depletion, the expression of an ATPase-deficient dominant-negative mutant of VPS4A (VPS4A^E228Q^) resulted in the accumulation of MIL^+^ structures under both starved and non-starved conditions (Fig. 6a, b). Moreover, VPS4A^E228Q^ signals were frequently detected on MIL^+^MPL^−^ phagophore-like structures (Fig. 6c–f) and the OAM of MIL^+^MPL^+^ nascent autophagosome-like structures (Fig. 6g). Quantification analysis revealed that nearly 80% of VPS4A^E228Q^ signals were associated with MIL^+^ immature autophagosomal membranes (Fig. 6h). Since CHMP2A depletion or VPS4 inhibition strongly accumulated phagophores even under non-starved condition, impairment of the ESCRT machinery might not only inhibit phagophore closure but also enhance autophagosome biogenesis. To explore this possibility, we measured autophagic flux upon the expression of VPS4A^E228Q^. In comparison to control GFP transfected cells, the protein levels of LC3-II and p62 were increased in VPS4A^E228Q^-transfected cells but did not further increase upon lysosomal inhibition with BafA1 (Fig. 6i, lanes 2, 5, 6), suggesting that disruption of the ESCRT machinery impacts autophagy flux rather than induction. Indeed, VPS4 inhibition had minimal effect on the induction of basal autophagy as the levels of LC3-II-increase during the 6 h period of BafA1 treatment were comparable between GFP and VPS4A^E228Q^ transfectants, indicating that VPS4 inhibition does not affect LC3-I to LC3-II conversion. Collectively, these results demonstrate that VPS4 activity is required for phagophore closure.

### Functional autolysosome formation requires membrane closure

**Fig. 7:**
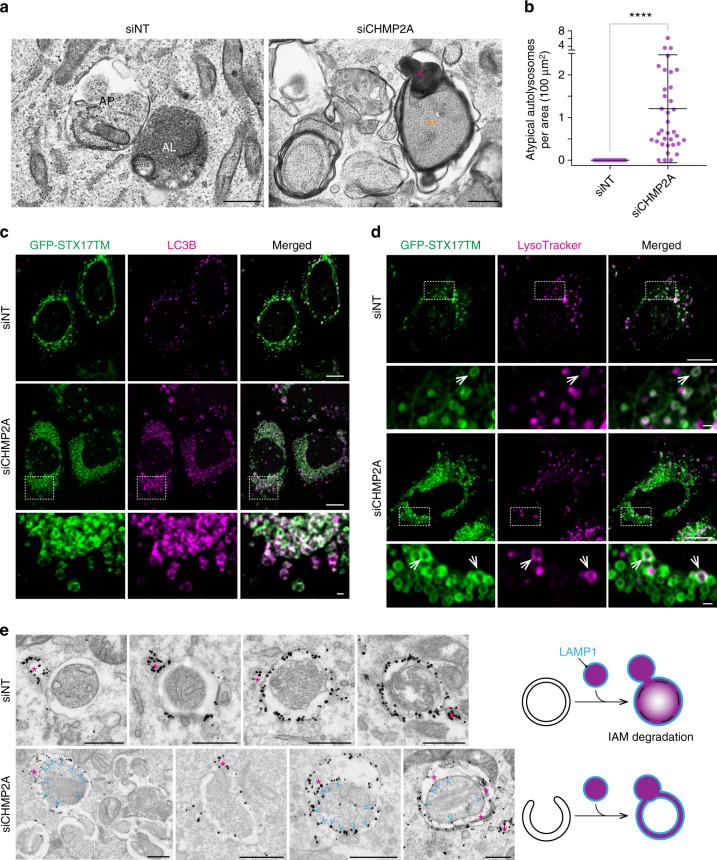
Phagophore closure is a critical step in functional autolysosome formation. **a** Typical images of atypical autolysosome-like structures in CHMP2A-depleted cells and normal autophagosome (AP)-like and autolysosome (AL)-like structures in starved, control siNT-transfected cells are shown. Asterisks and double-asterisks indicate lysosomal (and endolysosomal) contents and IAM-intact autophagosome-like structures, respectively. **b** The number of atypical autolysosomes per cytoplasmic area in (**a**) was quantified and shown (*n* = 35). Statistical significance was determined by Mann–Whitney nonparametric t-test. All values are mean ± SD. ****p ≤ 0.0001. U-2 OS cells stably expressing GFP-STX17TM (**c**, **d**) or wild-type U-2 OS cells (**e**) were transfected with the indicated ON-TARGETplus SMART Pool siRNAs for 48 h. **c** Cells were starved for 2 h, stained for endogenous LC3B, and subjected to confocal microscopy. **d** Cells were starved for 1.5 h, incubated with 100 nM LysoTracker Deep Red, and subjected to confocal microscopy. **e** Cells were starved for 3 h and subjected to immunoelectron microscopy using anti-LAMP1 antibody. Asterisks and arrowheads indicate lysosomal/endosomal LAMP1-positive structures and LAMP1 signals on the IAM, respectively. The scale bars represent 1 μm in (**a**), 10 μm in (**c, d**), 1 μm in the magnified images in (**c, d**), and 0.5 μm in (**e**)

Despite impaired autophagosome completion, the number of MPL^+^ structures were slightly but significantly increased by CHMP2A depletion or VPS4 inhibition (Figs. 3b, 6b). Interestingly, electron dense lysosomal structures were occasionally observed between the outer and inner membrane space of autophagosome-like structures in CHMP2A-depleted cells (Fig. 7a, b; Supplementary Fig. 6c). These structures are quite different from a typical autolysosome, in which the IAM is digested to distribute electron dense lysosomal material throughout the vacuole (left panel in Fig. 7a). Notably, we found that CHMP2A depletion did not block the recruitment of the autophagosome-lysosome fusion mediator, syntaxin 17 (STX17)^27^, to LC3-positive membranes (Fig. 7c). Moreover, while the majority of the STX17-positive structures in CHMP2A-depleted cells were negative for LysoTracker, a small portion of the autophagic structures were labelled by LysoTracker (Fig. 7d). These results suggest that lysosomal fusion can still proceed in the absence of autophagosome completion. However, in stark contrast to control cells where LysoTracker was primarily diffuse in the vacuole lumen, the LysoTracker signals in CHMP2A-depleted cells formed ring-shaped structures (arrows in Fig. 7d). This is similar to that reported in ATG3-deficient cells^17^ and suggests the impairment of IAM degradation to form a functional autolysosome.

As the inhibition of phagophore closure prevents the separation of the OAM from the IAM, aberrant fusion of an unclosed autophagosomal membrane with lysosomes/late endosomes would result in the distribution of glycosylated lysosomal membrane proteins throughout the autophagosomal membrane. To determine if this occurs in CHMP2A-depleted cells, we performed IEM using an anti-lysosome-associated membrane glycoprotein 1 (LAMP1) antibody. LAMP1 signals were detected on the OAM, but not the IAM, in control siNT-transfected cells (Fig. 7e; Supplementary Fig. 9), indicating that lysosomal fusion occurs after the completion of autophagosome formation. In contrast, while the majority of autophagic structures in CHMP2A-depleted cells were negative for LAMP1, some of the phagophore/immature autophagosome-like structures were found to be LAMP1 positive and contained LAMP1 signals in both OAM and IAM (Fig. 7e; Supplementary Fig. 9), indicating the importance of phagophore closure for the proper autolysosome formation.

**Fig. 8:**
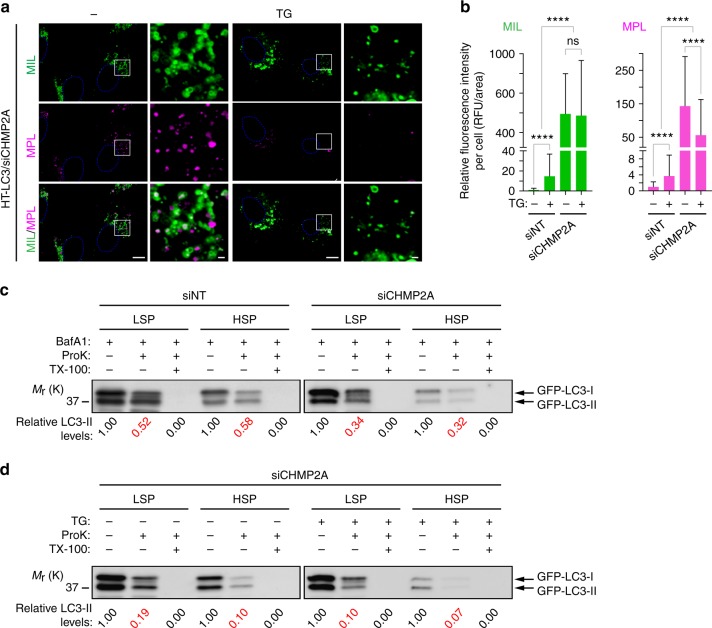
The SERCA inhibitor thapsigargin further accumulates unclosed autophagosomal membranes in CHMP2A-depleted cells. HT-LC3- (**a**, **b**) and GFP-LC3-expressing (**c, d**) U-2 OS cells were transfected with the indicated ON-TARGETplus SMART Pool siRNAs for 48 h. The indicated inhibitors (100 nM thapsigargin (TG); 100 nM BafA1) were added in the last 18 h of culture. **a** Cells were subjected to the HT-LC3 autophagosome completion assay followed by confocal microscopy. The scale bars represent 10 μm and 1 μm in the magnified images. **b** The cytoplasmic fluorescence intensities of MIL and MPL per cell in (**a**) were quantified and normalized to the respective mean fluorescence intensities of control siNT-transfected cells (*n* > 130). Data shown are representative of two independent experiments. Statistical significance was determined by Kruskal–Wallis one-way ANOVA on ranks followed by Dunn’s multiple comparison test. All values are mean ± SD. ns not significant; ****p ≤ 0.0001. **c**, **d** The low-speed pellet (LSP) and high-speed pellet (HSP) prepared from the postnuclear supernatant were resuspended in homogenate buffer in the presence or absence of proteinase K (ProK) and Triton X-100 (TX-100) and subjected to the proteinase K protection assay followed by immunoblotting using an anti-LC3 antibody. The LC3-II levels were quantified and normalized to the respective non-treatment control

Finally, we determined if the atypical phagophore-lysosome fusion contributes to the increase in MPL^+^ structures upon CHMP2A depletion. To this end, cells were depleted of CHMP2A in the presence or absence of the sarco/ER Ca2^+^ ATPase (SERCA) inhibitor thapsigargin (TG), which has been shown to arrest autophagy by blocking autophagosome fusion with endosomes and lysosomes^28^. As expected, we found that MPL but not MIL signals were significantly decreased in siCHMP2A-transfected cells while TG increased both MIL and MPL in control cells (Fig. 8a, b). To verify if the TG-induced MPL signal reduction is due to the inhibition of atypical phagophore closure in CHMP2A-depleted cells, we performed the GFP-LC3 protease protection assay^29^, which is based on the accessibility of protease to autophagosome-sequestered (inaccessible) and autophagosome-unsequestered (accessible) LC3. Consistent with the HT-LC3 assay results, we found that the sensitivity of GFP-LC3-II to proteinase K (ProK) was enhanced by CHMP2A depletion in both low-speed and high-speed pellet (HSP) fractions (Fig. 8c), and that TG treatment further increased the proportion of protease-accessible GFP-LC3-II in CHMP2A-depleted cells (Fig. 8d). Collectively, these results further demonstrate the role of CHMP2A in proper phagophore closure during autophagosome biogenesis.

## Discussion

The ESCRT components have been suggested as potential regulators of autophagosomal membrane closure^7,12,13^. However, characterization of the roles of each ESCRT protein in phagophore closure is technically challenging due to the lack of a robust assay that can distinguish phagophores and nascent autophagosomes. In this study, we develop an HT-LC3 autophagosome completion assay and demonstrate a role for ESCRT proteins in phagophore closure. By sequentially labelling membrane-unenclosed and membrane-enclosed HT-LC3-II with saturated doses of MIL and MPL, respectively, we are able to distinguish MIL^+^MPL^−^ phagophores, MIL^+^MPL^+^ nascent autophagosomes, and MIL^−^MPL^+^ mature autophagic vacuoles. We demonstrate that our assay provides a superior signal-to-noise ratio and high reproducibility with a semi-quantitative output and high-throughput adaptability by performing a siRNA screen of the ESCRT components and identifying several subunits, including the ESCRT-III CHMP2A, as critical regulators of phagophore closure. As far as we know, this is the first experimental demonstration that phagophore closure is regulated by the ESCRT machinery.

Our data shows that CHMP2A translocates to the phagophore during autophagy. The observation that CHMP2A depletion dramatically increases MIL^+^MPL^−^ signals in the HT-LC3 autophagosome completion assay is consistent with the electron microscopy and proteinase K protection assay results that demonstrate the accumulation of unclosed phagophore-like structures and protease-unprotected GFP-LC3-II, respectively. The accumulation of MIL^+^MPL^−^ signals also occurs upon the inhibition of VPS4 by expression of the ATPase-deficient mutant VPS4A^E228Q^. Moreover, VPS4A^E228Q^ accumulates on MIL^+^MPL^−^ structures, suggesting that VPS4 functions together with CHMP2A at the phagophore closure site to drive the membrane fission and generate the OAM and IAM. While these results indicate the role of the ESCRT-mediated membrane abscission and subsequent OAM and IAM separation for phagophore closure, it has been proposed that impairment of MVB formation by ESCRT inactivation may cause metabolic stresses, which trigger pro-autophagic signaling^13^. Indeed, it has been reported that ESCRT defects activate JNK (a potent autophagy inducer^30^) in *Drosophila*^31,32^ and increase autophagic flux in *C. elegans*^33^. Consistently, although we find that the autophagic flux is inhibited by CHMP2A depletion and that the rate of LC3-I to LC3-II conversion is not enhanced by VPS4 inhibition, we observe slight accumulation of either or both of MIL and MPL signals in about one third of the cells transfected with siRNAs against other ESCRT components as compared to control siNT-transfected cells. Therefore, an elevated rate of autophagy induction due to the impairment of MVB formation may partially contribute to the drastic accumulation of phagophores in CHMP2A/VPS4-inactivated cells in addition to phagophore closure impairment.

While the current study focuses on the function of CHMP2A in autophagy, we also find other ESCRT-III proteins including CHMP3 and CHMP7 as potential regulators of phagophore closure. However, unlike CHMP2A, knockdown of these proteins only moderately accumulates MIL^+^ immature autophagosome-like structures and many of the other components of the ESCRT machinery appear to be dispensable for phagophore closure. This observation is not surprising, since it has previously been reported that, unlike the canonical ESCRT-mediated membrane fission requiring ESCRT-0, -I, -II, and –III subunits, only CHMP2 and CHMP4 are found to be critical ESCRT-III components during HIV budding^34^. Moreover, it has recently been shown that nuclear envelope reformation is regulated by the ESCRT-III subunits CHMP2A and CHMP7 in a manner that is independent of canonical upstream targeting and bridging molecules^35–37^. Thus, the phagophore closure may also be regulated by a noncanonical ESCRT pathway. Moreover, as the present siRNA screening only targets single ESCRT genes, it is important in the future to examine the effects of combinational targeting of functionally redundant ESCRT subunits (e.g., VPS4A/B, CHMP4A-C, and ALIX/ESCRT-I/II) on phagophore closure to fully characterize the ESCRT machinery for autophagosome completion.

Our data also show that, while phagophore closure is important for lysosome recruitment and fusion, a small fraction of unclosed autophagosomal membranes still can undergo lysosomal fusion. However, this event appears to be nonproductive, as LAMP1 is distributed on both the OAM and IAM and the lysosomal contents are accumulated in the intermembrane space rather than the lumen of the autophagosome-like structure. Moreover, the observation that nearly all of phagophore/immature autophagosome-like structures in CHMP2A-depeleted cells are positive for STX17 is consistent with a recent report showing that STX17 dissociation is triggered by IAM degradation^17^. We hypothesize that the generation of the OAM and IAM by ESCRT-mediated membrane fission prior to lysosomal fusion is critical to prevent the misdistribution of glycosylated lysosomal membrane proteins on the IAM, and the failure of which limits access to lysosomal proteases and lipases thus impairing degradation.

## Methods

### Reagents

The following antibodies were used for IB, immunofluorescence (IF) and IEM: ATG7 (IB; Santa Cruz, SC-8668, 1:300); ATG9A (IF; Cell Signaling, 13509, 1:300); ATG16L (IF, MBL, PM040, 1:400); β-Actin (IB, Sigma-Aldrich, A5441, 1:10,000); CD107a/LAMP1 (IEM, BD Biosciences, 555798, 1:100); CEP55 (IB; Santa Cruz, SC- 377018, 1:200); CHMP2A (IB, Proteintech, 104771-AP, 1:1,000); CHMP2B (IB, Cell Signaling, 76173, 1:1,000); CHMP3 (IB; Santa Cruz, SC-166361, 1:200); CHMP7 (IB; Santa Cruz, SC- 271805, 1:200); GFP (IEM, Abcam, ab6556, 1:500); MAP1LC3B (IB, Novus, NB100-2220, 1:3,000; IF, Cell Signaling, 3868, 1:200); Nanogold- Fab’ (IEM; Nanoprobes, 2002 (mouse), 2004 (rabbit), 1:500); p62 (IB, IF; American Research Products, 03-GP62-C, 1:4,000 (IB), 1:400 (IF)). Accell SMART Pool siRNAs and ON-TARGETplus SMART Pool siRNAs listed in table S1 were purchased from GE Healthcare Dharmacon. All other reagents were obtained from the following sources: Bafilomycin A1 (LC Laboratories, B-1080); bovine serum albumin (BSA) (EMD Millipore, 126575; Calbiochem, 126575 (for IEM)); digitonin (Sigma-Aldrich, D141); gelatin from cold water fish skin (Sigma-Aldrich, G7765); GoldEnhance EM (Nanoprobes, 2113); Membrane-impermeable HaloTag Ligand (MIL) (Promega, Alexa Fluor 488-conjugated, G1001; Alexa Fluor 660-conjugated, G8471); Membrane-permeable HaloTag Ligand (MPL) (Promega, tetramethylrhodamine-conjugated, G8251); normal goat serum (Sigma-Aldrich, G9023); Nucleofector Kit V (Lonza, VCA-1003); Nucleofector Kit R (Lonza, VCA-1001); paraformaldehyde (Electron Microscopy Sciences, 15710); proteinase K (Invitrogen, 25530-049); saponin (Sigma-Aldrich, 84510); thapsigargin (Sigma-Aldrich, T9033); XF Plasma Membrane Permeabilizer (XF-PMP) (Seahorse Bioscience, 102504-100). pHaloTag-human MAP1LC3-Lv110 (HT-LC3) was custom-made by GeneCopoeia. The human CHMP2A cDNA (gift from Dr. Gerlich, Addgene#31805) was amplified by PCR using a primer set (5′–TTTGCTAGCGCCACCATGGACCTATTGTTCGGGC-3′; 5′-TTGAATTCGGTCCCTCCGCAGGTTCTTAA-3′) and subcloned into the Nhe I-EcoRI site of pCDH1-EGFP(N1)-EF1-puro. The linker sequence between CHMP2A and GFP is N′-(CHMP2A)-RILQSTVPRARDPPVAT-(GFP)-C′. The pCDH1-EGFP(N1)-EF1-puro vector was generated by subcloning the MCS-EGFP sequence of pEGFP-N1 (Clontech, #6085-1) into the Nhe I-NotI site of pCDH1-MCS1-EF1-Puro (System Biosciences, #CD510A-1). The following plasmids were gifted from Dr. Mizushima: pMXs-IP-EGFP-LC3 (Addgene#38195);^38^ pMXs-IP-EGFP-mAtg5 (Addgene#38196);^38^ pMRXIP GFP-STX17TM (Addgene#45910)^27;^ pEGFP-C1-human ATG2A (Addgene#36456)^29^. pCDH1-EGFP-human ATG2A, lentiCRISPR v2-human ATG2A sgRNA, pLX-human ATG2B sgRNA, lentiCRISPR v2-human ATG7 sgRNA were generated as previously described^39^. Human ATG13 sgRNAs (5′-TCTTTTCACCAAGCCGAGCC-3′, 5’-CACATGGACCTCCCGACTGC-3′, and 5′-CAGTCTGTTGTACACCGTGT-3′) were sub-cloned into LRG (Lenti_sgRNA_EFS_GFP) (Addgene 65656).

### Cell culture

HeLa cells and U-2 OS cells were obtained from American Type Culture Collection and maintained in Dulbecco’s Modification of Eagle’s Medium (DMEM) and McCoy’s 5 A Medium, respectively, supplemented with 10% fetal bovine serum and 1 × Antibiotic Antimycotic Solution (Corning, 30-004-CI).

### Transfection and viral transduction

Retrovirus-mediated and lentivirus-mediated gene transductions were performed as described previously^40^. To generate HT-LC3 U-2 OS and HeLa cells, cells were transduced with lentiviruses encoding HT-LC3 and selected with 1 µg/ml puromycin for 5 days. ATG2A/B double-knockout and ATG7 knockout U-2 OS cells were generated as previously described^39^. To generate ATG13 knockout U-2 OS cells, cells were transfected with an equal amount (6 µg each per a 10-cm dish) of three human ATG13 gRNA for 48 h and sorted for GFP-positive transfected cells. Fourteen days after transfection, the cells were re-sorted for GFP-negative population to eliminate Cas9 stable transfectants and used for experiments. For siRNA screening, cells were grown overnight on the Lab-TekII 8-well Chambered Coverglass (Nunc, 155409) and incubated in Accell siRNA Delivery Medium (Dharmacon, B-005000-100) containing 1 µM Accell siRNA for 72 h. siRNA-mediated gene silencing was performed by nucleofection according to the manufacture’s protocol.

### Fluorescence microscopy and electron microscopy

Cells were grown on Lab-TekII Chambered Coverglass, Chamber Slide (Nunc, 154941) or Glass Bottom Dish (MatTek, P35GCOL-0-14-C). IF was performed as follows: for the detection of p62 and ATG9A, cells were fixed in 4% paraformaldehyde (PFA)-phosphate-buffered saline (PBS) for 10 min and permeabilized with 0.1% Triton X-100 for 3 min. For LC3, cells were permeabilized and fixed in methanol at −20 °C for 10 min. Cells were then incubated in 10% normal goat serum for 1 h followed by the primary and the secondary antibodies and mounted with ProLong Gold Antifade Mountant (Thermo Scientific, P10144 or P36941 (with DAPI)). Fluorescent images were obtained using a Leica AOBS SP8 laser-scanning confocal microscope (63× water or oil-immersion lens), or an OLYMPUS IX81 deconvolution microscope (63× oil-immersion lens), deconvolved using Huygens deconvolution software (Scientific Volume Imaging) or SlideBook software (Intelligent Imaging Innovations), and analyzed using Imaris software (Bitplane), Volocity software (PerkinElmer) or SlideBook software. Electron microscopy was performed as previously described^41,42^. Briefly, cells were grown on Thermanox plastic coverslips (Thermo Scientific, 174950) overnight, incubated in SM for 2–3 h, fixed in 2% paraformaldehyde-2.5% glutaraldehyde in 0.1 M cacodylate buffer, pH 7.3, for 1.5 h at room temperature followed by post-fixation buffer (1% osmium tetroxide/1.5% potassium ferrocyanide-0.1 M sodium cacodylate, pH 7.3) for 1 h or overnight, dehydrated in a graded series of ethanol, embedded in EMbed 812 resin (Electron Microscopy Sciences, 14120), sectioned at a thickness of 70 nm, mounted on mesh copper grids, stained with aqueous uranyl acetate and lead citrate and analyzed using a JEOL JEM 1400 transmission electron microscope. For the quantification of autophagic structures, samples were post-fixed in the absence of potassium ferrocyanide.

### Immunoelectron microscopy

IEM was performed as described previously^43,44^. Briefly, cells grown on Thermanox plastic coverslips were starved for 2–3 h, fixed in 4% paraformaldehyde-phosphate buffer (pH 7.4) (PB) for 2 h, permeabilized with 0.25% saponin for 30 min, incubated in blocking buffer (10% BSA, 10% normal goat serum, 0.1% cold water fish gelatin, 0.1% saponin) for 30 min followed by the primary (overnight at 4 °C) and the secondary (for 1 h) antibodies, post-fixed in 1% glutaraldehyde-PB for 10 min, and washed in 50 mM glycine-PBS for 15 min. The signals were then intensified with the GoldEnhance EM for 2–3 min. After washing in 1% sodium thiosulfate for 5 s, the specimens were incubated in post-fixation buffer (1% osmium tetroxide/1.5% potassium ferrocyanide-0.1 M sodium cacodylate, pH 7.3) and processed as described above.

### HaloTag-LC3 autophagosome completion assay

HaloTag-LC3 expressing cells were incubated in 1× MAS buffer (220 mM mannitol, 70 mM sucrose, 10 mM KH_2_PO_4_, 5 mM MgCl_2_, 2 mM HEPES, 1 mM EGTA) containing XF-PMP (2–3 nM for U-2 OS and 3 nM for HeLa cells) and MIL at 37 °C for 15 min. Alternatively, cells were permeabilized with 20 μM digitonin at 37 °C for 2 min and incubated with MIL at 37 °C for 15 min (Fig. 1h-j; Supplementary Fig. 1c). Cells were then fixed in 4% PFA for 5 min, washed three times in PBS, and incubated with MPL for 30 min. After washing three times in PBS, cells were analyzed by fluorescence deconvolution or confocal microscopy.

### Correlative light electron microscopy

For correlative light electron microscopy (CLEM), cells were grown overnight on Gridded Glass Bottom Dish (MatTek, P35G-1.5-14-C-GRID), starved for 2 h, fixed in 4% PFA-PBS for 5 min, and incubated with 1× MAS containing XF-PMP and MIL for 30 min followed by MPL for 30 min. Cells of interest were identified by correlating the grid, and three-dimensional images were obtained by confocal microscopy before processing for electron microscopy.

### Immunoblotting

Total cell lysates were prepared in radio-immunoprecipitation assay buffer (150 mM NaCl, 10 mM Tris-HCl, pH 7.4, 0.1% SDS, 1% Triton X-100, 1% Deoxycholate, 5 mM EDTA, pH 8.0) containing protease and phosphatase inhibitors and subjected to SDS-PAGE followed by IB with the indicated antibodies. The signal intensities were quantified using the Image Studio version 5 software (LI-COR Biotechnology)^45^. Uncropped scans of all the blots are shown in Supplementary Fig. 10.

### Protease protection assay

Cells were resuspended in ice-cold homogenization buffer (HB: 0.25 M sucrose, 140 mM NaCl, 1 mM EDTA, 20 mM Tris-HCl, pH8.0), passed 10 times through a 27-guage syringe needle and then centrifuged at 300 × *g* at 4 °C for 5 min to obtain post-nuclear supernatant (PNS). The PNS was centrifuged at 7600 × *g* at 4 °C for 5 min to obtain low-speed pellet (LSP) and supernatant (S76). The S76 was then centrifuged at 100,000 × *g* at 4 °C for 30 min to obtain high-speed pellet (HSP) and supernatant. Each pellet fraction was resuspended in ice-cold HB, equally divided into three tubes and incubated with or without 100 µg/ml proteinase K and 0.5% Triton X-100 on ice for 30 min. After the addition of 1 mM phenylmethylsulfonyl fluoride to stop the reaction, the reaction mixture was subjected to IB.

### Statistical analyses

Statistical significance was determined using Graph Pad Prism 7.0. Threshold for statistical significance for each test was set at 95% confidence (*p* < 0.05).

### Data availability

All the data that support the findings of this study are available on request from the corresponding authors (Y.Y; H.-G.W).

## Electronic supplementary material

Supplementary Information
