# Supplementary material for: An autophagy assay reveals the ESCRT-III component CHMP2A as a regulator of phagophore closure
Source: Nat Commun. 2018 Jul 20;9:2855. doi: 10.1038/s41467-018-05254-w (PMC6054611; doi:10.1038/s41467-018-05254-w)
Supplement: Supplementary file 1 — Supplementary Information [file 41467_2018_5254_MOESM1_ESM.pdf]

# Supplementary Information for

**An autophagy assay reveals the ESCRT-III component CHMP2A as a regulator of phagophore closure**

Yoshinori Takahashi,[1]* Haiyan He,[1] Zhenyuan Tang,[1] Tatsuya Hattori,[1] Ying Liu,[1] Megan M. Young,[1] Jacob M. Serfass,[2] Longgui Chen,[1] Melat Gebru,[1] Chong Chen,[1] Carson A. Wills,[1] Jennifer M. Atkinson,[1] Han Chen,[4] Thomas Abraham,[3, 4] Hong-Gang Wang,[1,2]*

[1] Department of Pediatrics, [2] Department of Pharmacology, [3] Microscopy Imaging Facility, [4] Department of Neural and Behavioral Science, Penn State College of Medicine, Hershey, PA 17033

*Corresponding authors. Email: ytakahashi@pennstatehealth.psu.edu; huw11@pus.edu

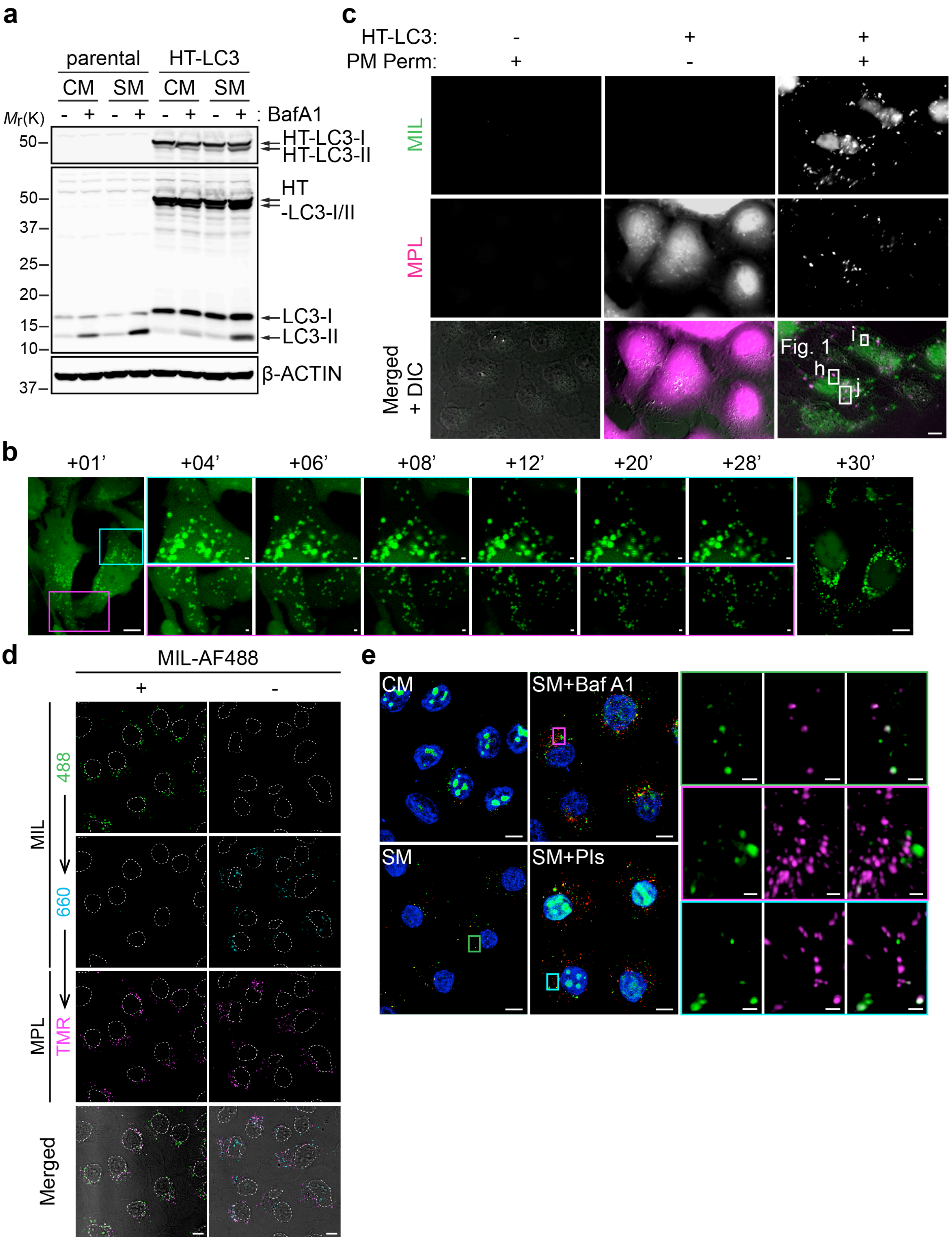

a
parental
HT-LC3
CM
SM
CM
SM
Mr(K)
: BafA1
50
HT-LC3-I
HT-LC3-II
50
37
25
20
15
10
HT
-LC3-I/II
LC3-I
LC3-II
37
β-ACTIN
b
+01'
+04'
+06'
+08'
+12'
+20'
+28'
+30'
c
HT-LC3:
PM Perm:
MIL
MPL
Merged + DIC
Fig. 1
h
i
j
d
MIL-AF488
MIL
488
660
MPL
TMR
Merged
e
CM
SM+Baf A1
SM
SM+PIs

**Supplementary Figure 1. Establishment of the HT-LC3 autophagosome completion assay. (a)** U-2 OS cells were transduced with lentiviruses encoding HT-LC3 and selected with puromycin for 5 days. The resultant stable transfectants and the parental wild-type cells were incubated in starvation medium (SM) or control complete medium (CM) in the presence or absence of 100 nM BafA1 for 3 h and subjected to immunoblotting using the indicated antibodies. **(b)** HT-LC3 U-2 OS cells were starved in the presence of 100 nM BafA1 for 1.5 h, stained with TMR-MPL, and treated in 1xMAS containing 3 nM XF-PMP at 37°C for the indicated periods of time (min). Fluorescence images were acquired every 1 min for 30 min using a Leica AOBS SP8 laser-scanning confocal microscope. Magnified images of the boxed areas are shown in the middle panels. **(c)** HT-LC3 U-2 OS and wild-type U-2 OS cells were starved for 2 h, permeabilized with digitonin (PM Perm) and subjected to the HT-LC3 autophagosome completion assay. The fluorescence images were obtained by 3D deconvolution microscopy. Magnified images of the boxed areas are shown in Fig. 1h-j. **(d)** HT-LC3 U-2 OS cells were starved for 3 h, incubated with 3 nM XF-PMP in the presence or absence of AF488-MIL at 37°C for 15 min, fixed, then incubated with AF660-MIL at RT for 30 min followed by TMR-MPL at RT for 30 min, and subjected to confocal microscopy. **(e)** HeLa cells stably expressing HT-LC3 were incubated in CM or SM in the presence or absence of lysosomal inhibitors (100 nM Baf A1 or protease inhibitors (PIs; 10 µg/ml pepstatin A, 1 µM leupeptin, 10 µM E64d)) for 2 h and subjected to the HT-LC3 autophagosome completion assay followed by confocal microscopy. The scale bars represent 10 µm and 1 µm in the magnified images in **b** and **e**.

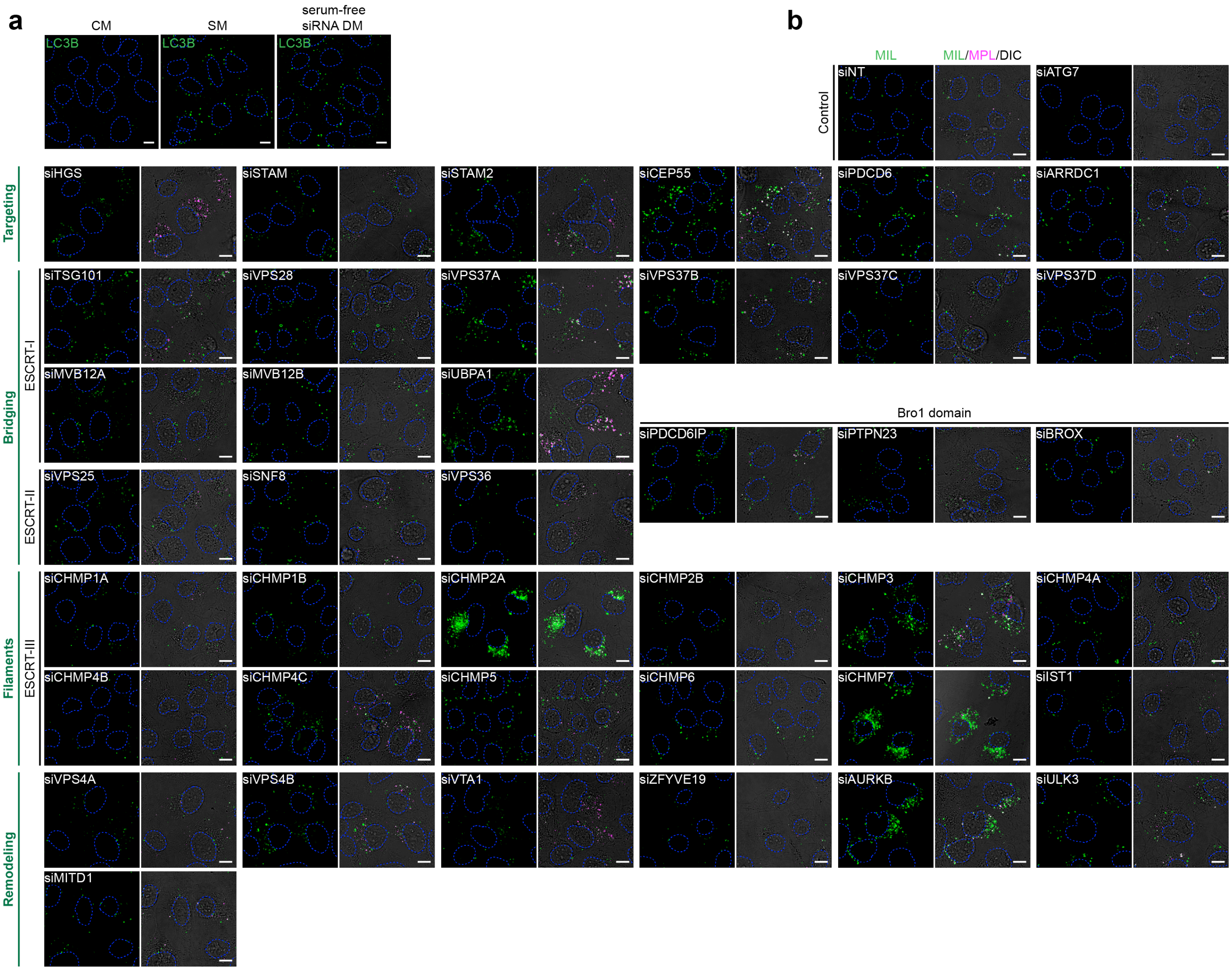


**Supplementary Figure 2. Screening of ESCRT components and their regulatory proteins using the HT-LC3 autophagosome completion assay. (a)** HT-LC3 U-2 OS cells were cultured in serum-free Accell siRNA delivery medium (DM) for 48 h or CM for 45 h followed by SM or CM for 3 h, subjected to the HT-LC3 autophagosome completion assay, and analyzed by confocal microscopy. **(b)** HT-LC3 U-2 OS cells were incubated with the indicated Accell SMRT Pool siRNAs for 72 h, subjected to the HT-LC3 autophagosome completion assay, and analyzed by confocal microscopy. The scale bars represent 10 µm. Data shown are representative of two independent experiments (at least 5 images were taken at 63x magnification at each experiment).

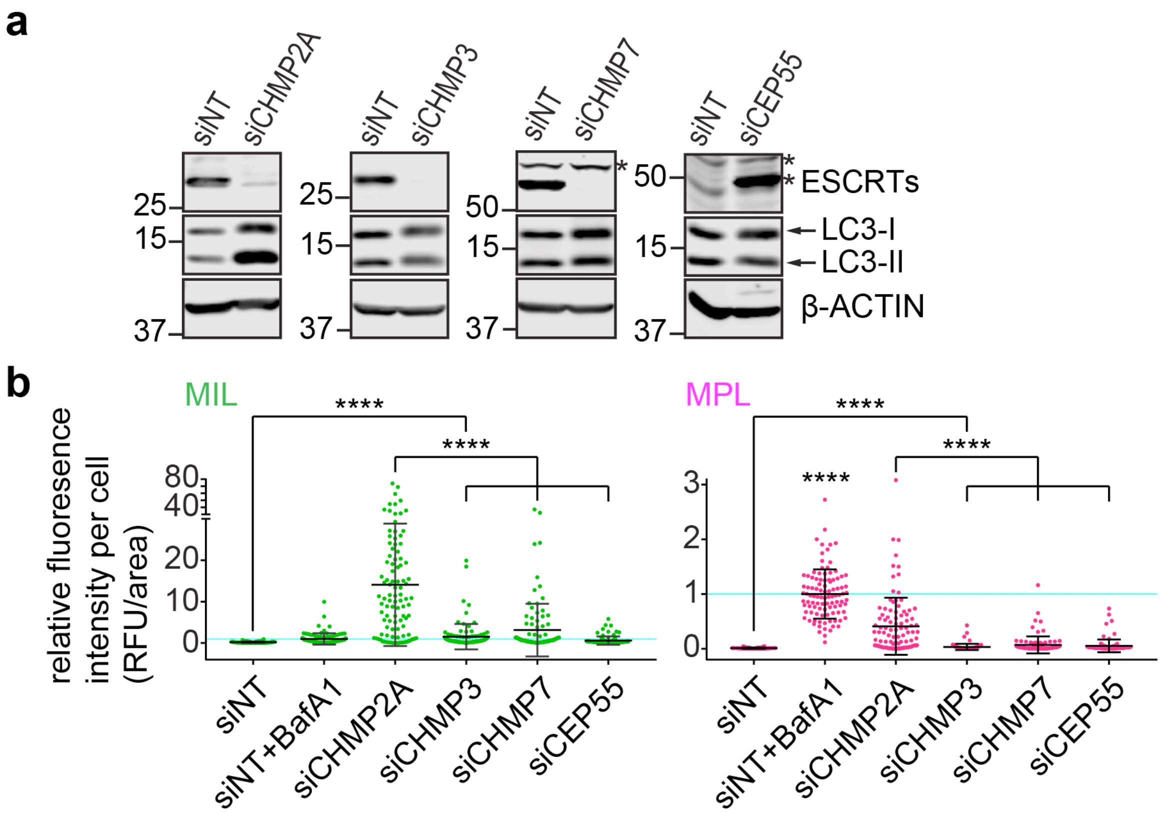


**Supplementary Figure 3. Depletion of ESCRT components leads to the accumulation of MIL$^{+}$.** HT-LC3 U2-OS cells were transfected with the indicated ON-TARGETplus SMART Pool siRNAs for 48 h and subjected to immunoblotting using the indicated antibodies **(a)** or starved for 3 h in the presence or absence of 100 nM BafA1 and subjected to the HT-LC3 autophagosome completion assay followed by confocal microscopy **(b)**. In **a**, the asterisks indicate non-specific bands. In **b**, the cytoplasmic fluorescence intensities of MIL and MPL in each cell were quantified and normalized to the respective mean fluorescence intensities of control siNT transfected cells starved in the presence of BafA1 ($n > 100$). Statistical significance was determined by Kruskal-Wallis one-way ANOVA on ranks followed by Dunn's multiple comparison test. All values are mean ± SD. ****$p \leq 0.0001$.

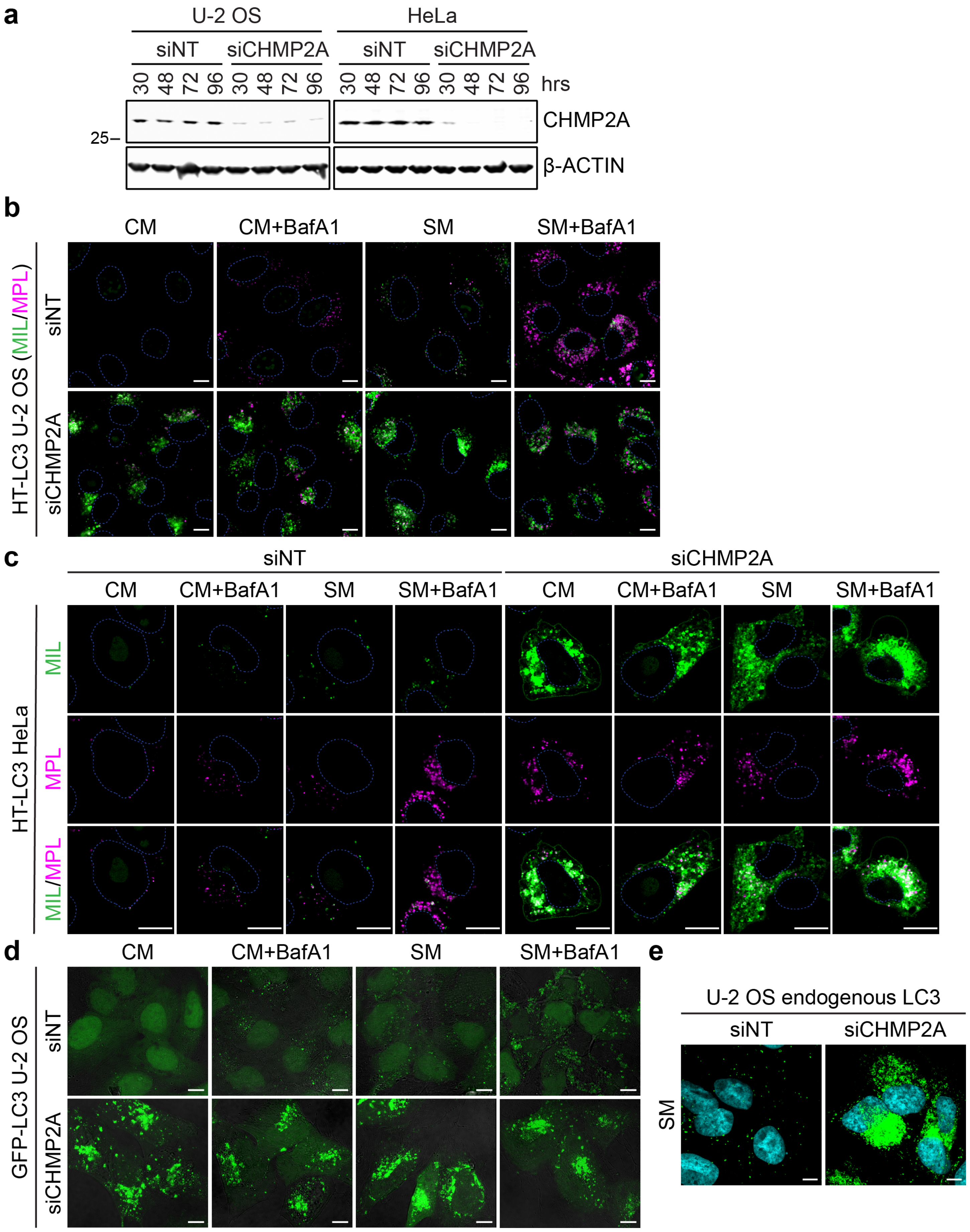
a
U-2 OS
HeLa
siNT
siCHMP2A
siNT
siCHMP2A
30 48 72 96 30 48 72 96 30 48 72 96 30 48 72 96 hrs
25–
CHMP2A
β-ACTIN
b
CM
CM+BafA1
SM
SM+BafA1
HT-LC3 U-2 OS (MIL/MPL)
siNT
siCHMP2A
c
siNT
siCHMP2A
CM
CM+BafA1
SM
SM+BafA1
CM
CM+BafA1
SM
SM+BafA1
HT-LC3 HeLa
MIL
MPL
MIL/MPL
d
CM
CM+BafA1
SM
SM+BafA1
GFP-LC3 U-2 OS
siNT
siCHMP2A
e
U-2 OS endogenous LC3
siNT
siCHMP2A
SM

**Supplementary Figure 4. Depletion of CHMP2A results in the accumulation of LC3-positive immature autophagosomal membranes. (a)** U-2 OS and HeLa cells were transfected with the indicated ON-TARGETplus SMART Pool siRNAs for the indicated periods of time and subjected to immunoblotting using the indicated antibodies. **(b, c)** HT-LC3 U-2 OS (**b**) or HT-LC3 HeLa (**c**) cells were transfected with the indicated siRNAs for 48 h, incubated in CM or SM in the presence or absence of 100 nM BafA1 for 2 h, and subjected to the HT-LC3 autophagosome completion assay. **(d)** U-2 OS cells expressing GFP-LC3 were transfected with the indicated siRNAs for 48 h and incubated in CM or SM in the presence or absence of 100 nM Baf A1 for 2 h. **(e)** Wild-type U-2 OS cells were transfected with the indicated siRNAs for 48 h, starved for 2 h, and subjected to immunofluorescence microscopy using anti-LC3B antibodies. All fluorescence images were obtained by confocal microscopy. The scale bars represent 10 μm.

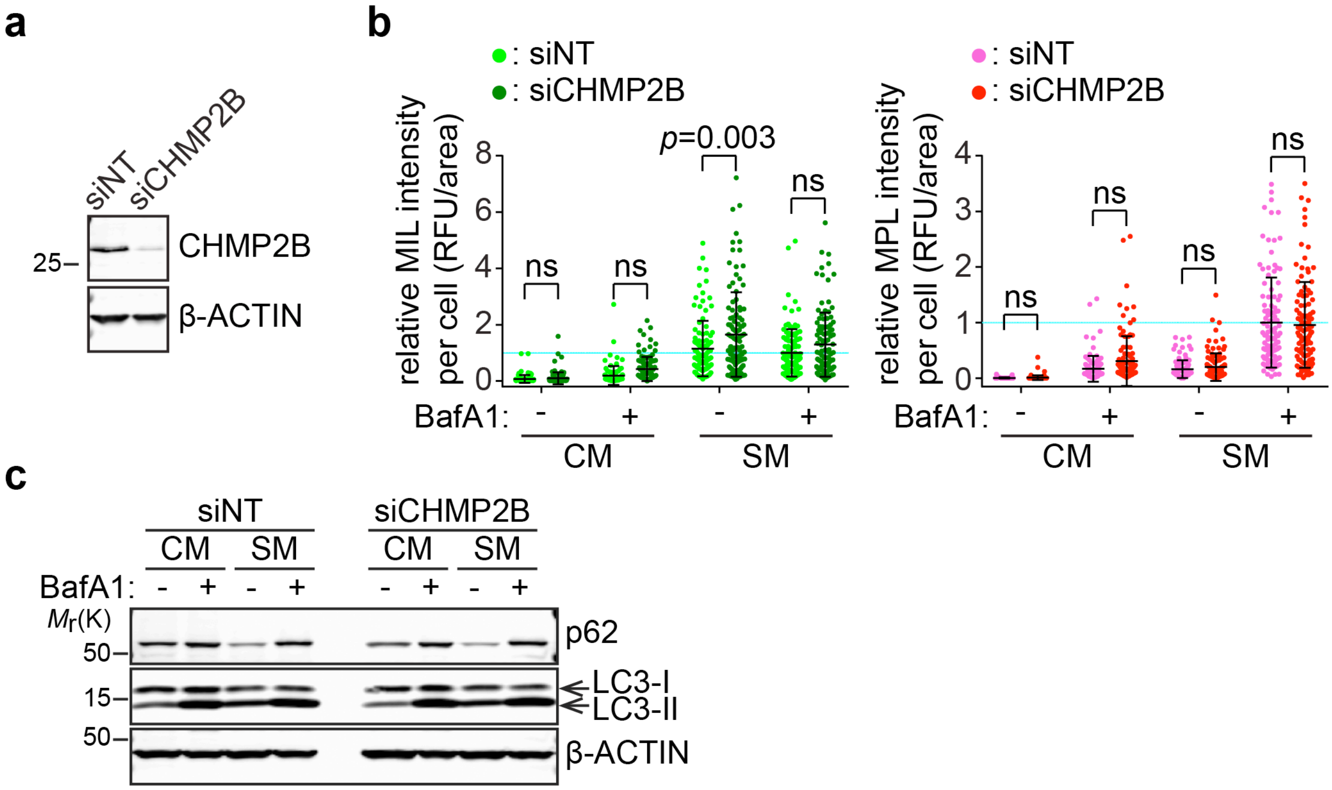


**Supplementary Figure 5. CHMP2B is dispensable for autophagosome formation.** Wild-type U-2 OS (**a**, **c**) or HT-LC3 U-2 OS (**b**) cells were transfected with the indicated ON-TARGETplus SMART Pool siRNAs for 48 h. **(a)** Cells were subjected to immunoblotting using the indicated antibodies. **(b)** Cells were incubated in CM or SM in the presence or absence of 100 nM Baf A1 for 2 h and subjected to the autophagosome completion assay followed by confocal microscopy. The cytoplasmic fluorescence intensities of MIL and MPL in each cell were quantified and normalized to the respective mean fluorescence intensities in the control siNT transfected cells starved in the presence of Baf A1 ($n$ > 100). The MIL/MPL ratio in each cell was calculated and shown at the right. Statistical significance was determined using two-way ANOVA with Sidak's multiple comparison test. All values are mean ± SD. ns not significant. **(c)** Cells were incubated in CM or SM in the presence or absence of 100 nM Baf A1 for 3 h and subjected to immunoblotting using the indicated antibodies.

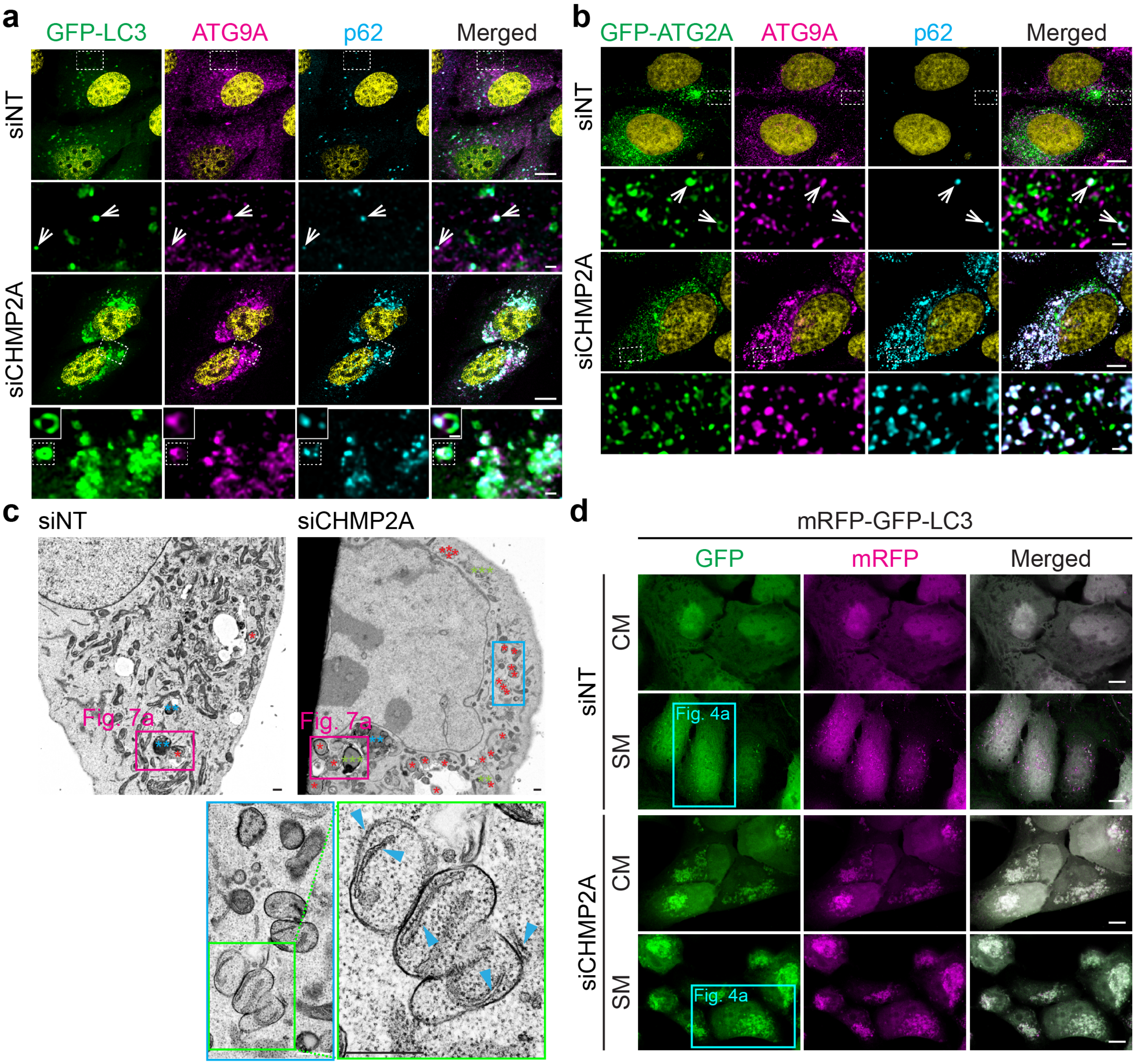


**Supplementary Figure 6. CHMP2A depletion accumulates ATG2, LC3, ATG9A, and p62-positive immature autophagosomal membranes and impairs autophagic flux. (a, b)** U-2 OS cells stably expressing GFP-LC3 (**a**) or ATG2A/B-deficient U-2 OS cells stably expressing GFP-ATG2A (**b**) were transfected with the indicated ON-TARGETplus SMART Pool siRNAs for 48 h, starved for 2 h, stained with the indicated antibodies, and subjected to confocal microscopy. Nuclei were stained with DAPI and shown as yellow. **(c)** Wild-type U-2 OS cells were transfected with the indicated siRNAs for 48 h, starved for 2 h, stained with the indicated antibodies, and subjected to electron microscopy. Red asterisks, blue asterisks, green asterisks, and arrowheads indicate oval-shaped phagophore-like structures,

autolysosomes, atypical autolysosomes, and phagophore-associated ER, respectively. Magnified images in the indicated areas are shown in Fig. 7a. **(d)** CHMP2A knockdown or control siNT transfected U-2 OS cells expressing mRFP-GFP-LC3 were starved for 3 h and subjected to confocal microscopy. Magnified images in the indicated areas are shown in Fig. 4a. The scale bars represent 10 μm and 1 μm in the magnified images.

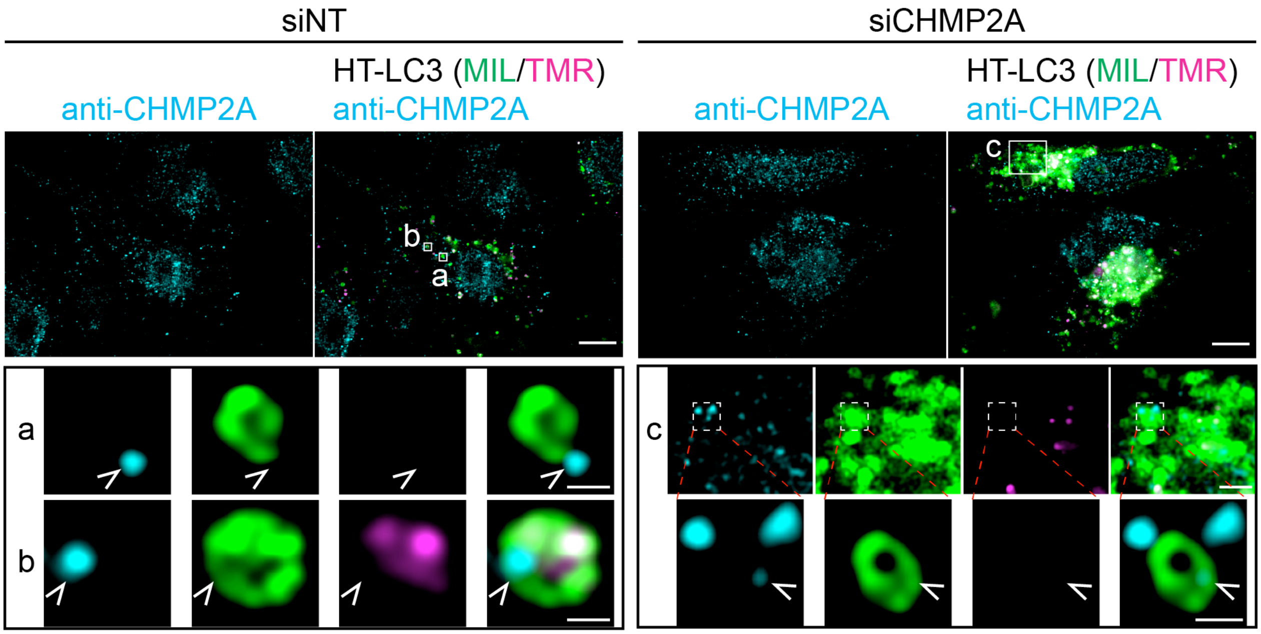


**Supplementary Figure 7. Immunofluorescence staining for endogenous CHMP2A during autophagy.** HT-LC3 U-2 OS cells were transfected with the indicated siRNAs, starved for 3 h, and subjected the HT-LC3 assay (AF660-MIL and TMR-MPL) followed by immunofluorescence with anti-CHMP2A antibodies (1:200, Proteintech, 104771-AP). Arrowheads indicate antibody signals on MIL+ immature autophagic structures. Magnified images of the boxed are shown in the lower panels. The scale bars represent 10 µm and 1 µm in the magnified images.

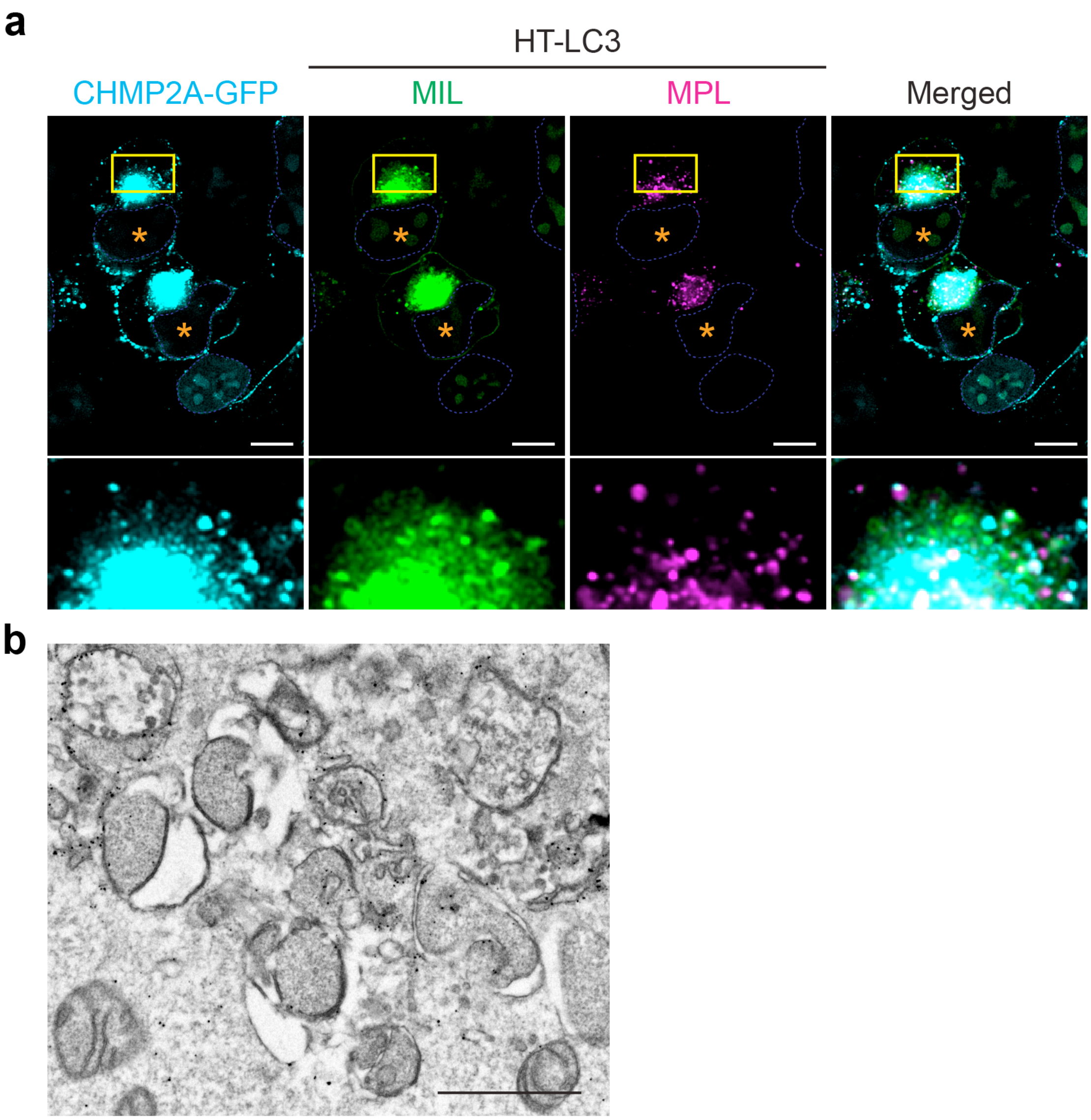


**Supplementary Figure 8. Overexpression of CHMP2A-GFP results in the accumulation of immature autophagosomal structures.** HT-LC3 U-2 OS cells were transiently transfected with CHMP2A-GFP for 24 h and subjected to the HT-LC3 autophagosome completion assay (**a**) and immunoelectron microcopy using anti-GFP antibody (**b**). The scale bars represent 10 µm in **a** and 1 µm in **b**.

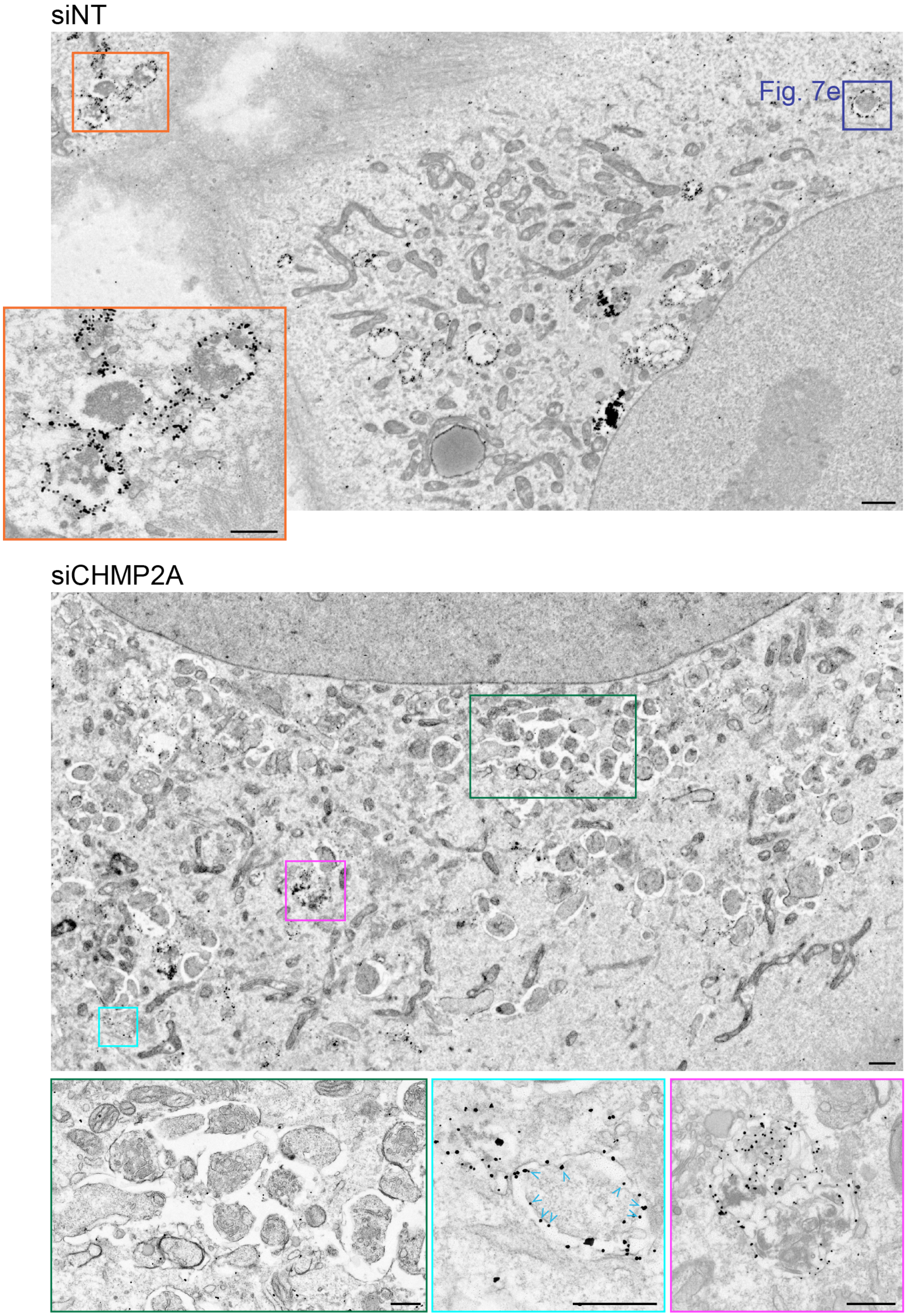


**Supplementary Figure 9. Immunoelectron micrographs of CHMP2A knockdown and control siNT-transfected, starved U-2 OS cells labelled with anti-LAMP1 antibody.** Wild-type U-2 OS cells were transfected with the indicated ON-TARGETplus SMART Pool siRNAs for 48 h, starved for 3 h, and subjected to immunoelectron microscopy using anti-LAMP1 antibody. Arrowheads indicate LAMP1 signals on the IAM. The scale bars represent 1 μm.

Fig. 4c

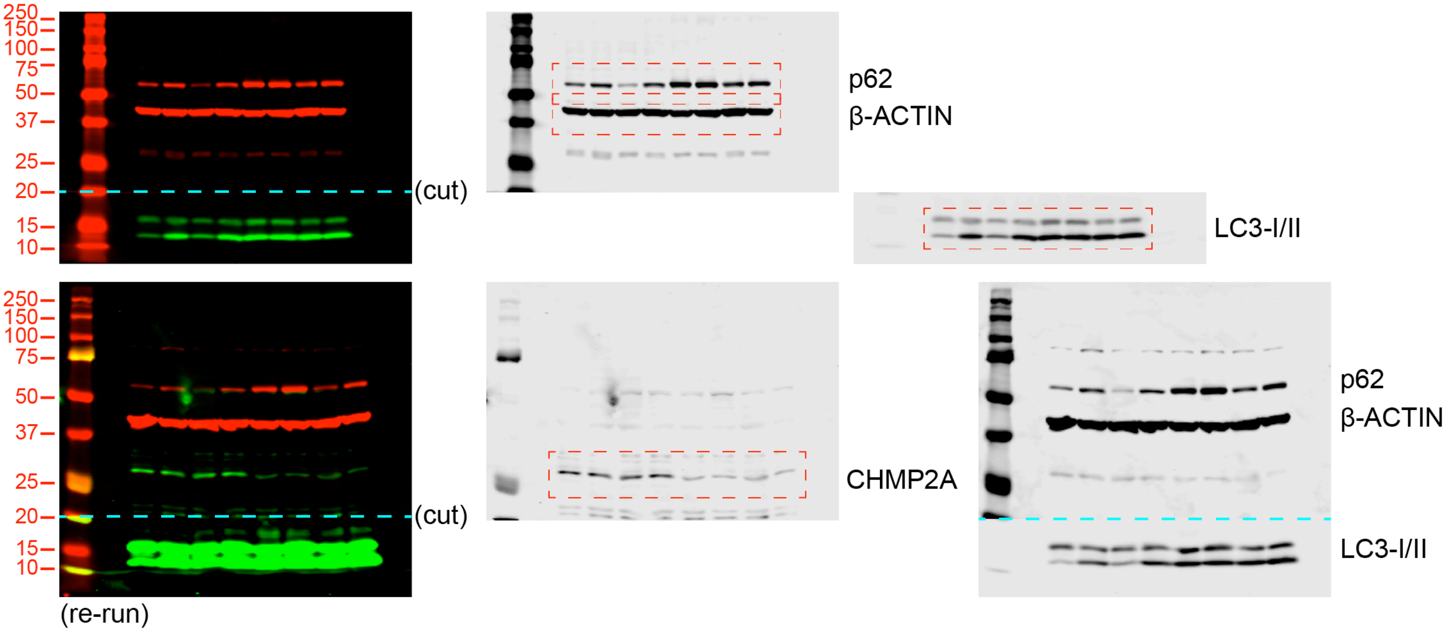


Fig. 6d

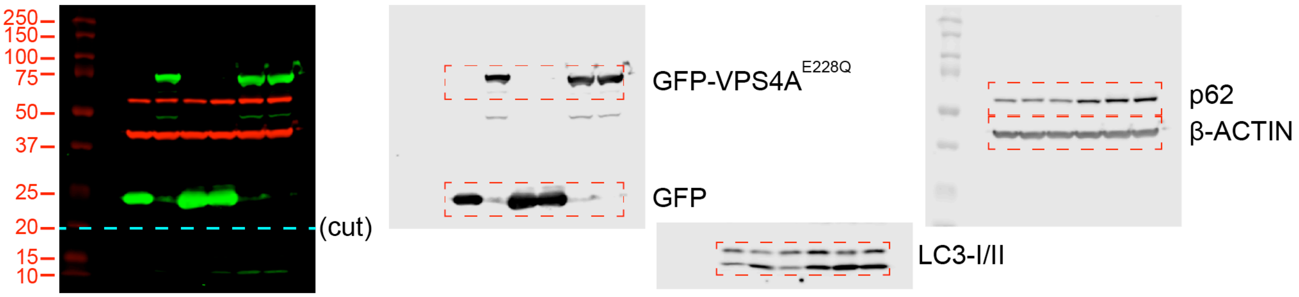


Fig. 8c

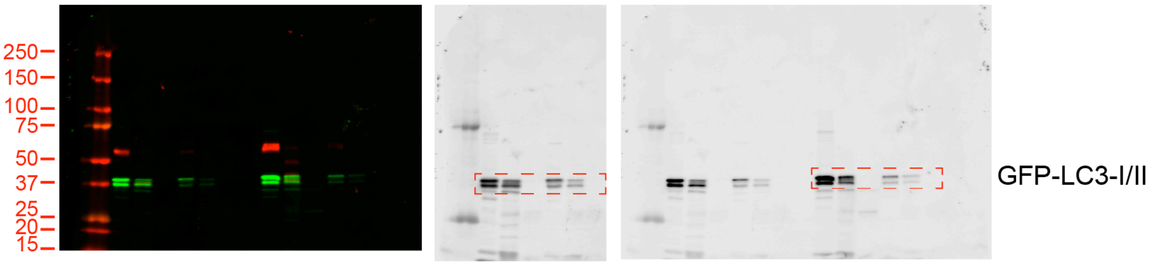


Fig. 8d

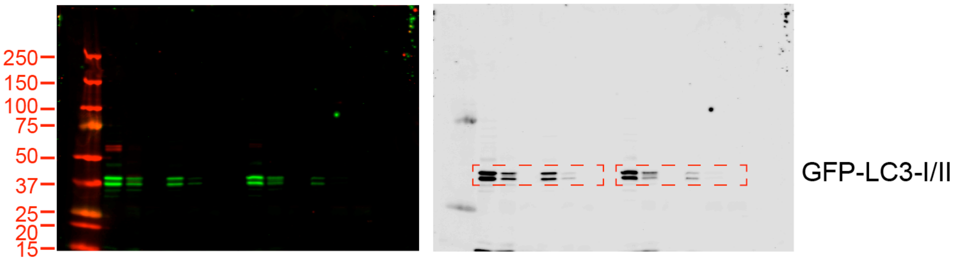


**Supplementary Figure 10. Uncropped scans of the most important blots.**

Supplementary Table 1. siRNAs used in this study.

| | | Gene Symbol | CatalogNumber |
|---|---|---|---|
| Accell SMART Pool siRNAs | **siRNA control** | Non-Targeting (NT) | E-001910-10 |
| | **negative control** | ATG7 | E-020112-00 |
| | **Targeting** | HGS | E-016835-00 |
| | | STAM | E-011423-00 |
| | | STAM2 | E-017361-00 |
| | | CEP55 | E-006893-00 |
| | | PDCD6 | E-004440-00 |
| | | ARRDC1 | E-015918-00 |
| | **Bridging (ESCRT-I)** | TSG101 | E-003549-00 |
| | | VPS28 | E-006486-00 |
| | | VPS37A | E-016816-00 |
| | | VPS37B | E-014404-00 |
| | | VPS37C | E-018204-00 |
| | | VPS37D | E-025253-00 |
| | | MVB12A | E-015563-00 |
| | | MVB12B | E-026207-00 |
| | | UBAP1 | E-017474-00 |
| | **Bridging (ESCRT-II)** | SNF8 | E-004695-00 |
| | | VPS25 | E-004699-00 |
| | | VPS36 | E-004701-00 |
| | **Bridging (Bro1)** | PDCD6IP | E-004233-00 |
| | | PTPN23 | E-009417-00 |
| | | BROX | E-015709-00 |
| | **Filaments (ESCRT-III)** | CHMP1A | E-010339-00 |
| | | CHMP1B | E-004698-00 |
| | | CHMP2A | E-020247-00 |
| | | CHMP2B | E-004700-00 |
| | | CHMP3 | E-004696-00 |
| | | CHMP4A | E-020698-00 |
| | | CHMP4B | E-018075-00 |
| | | CHMP4C | E-015932-00 |
| | | CHMP5 | E-004697-00 |
| | | CHMP6 | E-005060-00 |
| | | CHMP7 | E-015514-00 |
| | | IST1 | E-020977-00 |
| | **Remodeling** | VPS4A | E-013092-00 |
| | | VPS4B | E-013119-00 |
| | | VTA1 | E-016969-00 |
| | | ZFYVE19 | E-017961-00 |
| | | AURKB | E-003326-00 |
| | | ULK3 | E-004949-00 |
| | | MITD1 | E-016584-00 |
| **ON-TARGETplus SMART Pool siRNAs** | | Non-Targeting (NT) | D-001810-10 |
| | | CEP55 | L-006893-01 |
| | | CHMP2A | L-020247-01 |
| | | CHMP2B | L-004700-01 |
| | | CHMP3 | L-004696-00 |
| | | CHMP7 | L-015514-01 |
